# Cine Phase Contrast Magnetic Resonance Imaging of Calf Muscle Contraction in Pediatric Patients with Cerebral Palsy and Healthy Children: Comparison of Voluntary Motion and Electrically Evoked Motion

**DOI:** 10.3390/children13010116

**Published:** 2026-01-13

**Authors:** Claudia Weidensteiner, Xeni Deligianni, Tanja Haas, Philipp Madoerin, Oliver Bieri, Meritxell Garcia Alzamora, Jacqueline Romkes, Erich Rutz, Francesco Santini, Reinald Brunner

**Affiliations:** 1 Department of Radiology, Division of Radiological Physics, University Hospital Basel, 4031 Basel, Switzerlandfrancesco.santini@unibas.ch (F.S.); 2 Department of Biomedical Engineering, University of Basel, 4123 Allschwil, Switzerland; 3 Department of Radiology, University Hospital Basel, 4031 Basel, Switzerland; xeni.deligianni@ukbb.ch; 4 Paediatric Research Centre, University Children’s Hospital Basel, 4056 Basel, Switzerland; 5 Department of Radiology, Division of Neuroradiology, University Hospital Basel, 4031 Basel, Switzerland; meritxell.garciaalzamora@usz.ch; 6 Department of Neuroradiology, University Hospital Zürich, 8091 Zürich, Switzerland; 7 Centre for Clinical Motion Analysis, University Children’s Hospital Basel, 4056 Basel, Switzerland; j.romkes@unibas.ch; 8 Department of Orthopaedics, The Royal Children’s Hospital, Melbourne 3052, Australia; 9 Murdoch Children’s Research Institute, Melbourne 3052, Australia; 10 Department of Paediatrics, Bob Dickens Chair, Paediatric Orthopaedic Surgery, The University of Melbourne, Melbourne 3010, Australia; 11 Medical Faculty, University of Basel, 4001 Basel, Switzerland

**Keywords:** magnetic resonance imaging (MRI), cerebral palsy, skeletal muscle, motion, children, electrical muscle stimulation (EMS)

## Abstract

**Highlights:**

**What are the main findings?**

- Cine phase contrast MRI for time-resolved assessment of leg muscle function during voluntary and electrically evoked motion was feasible in typically developing children.
- The success rates of cine phase contrast MRI during both voluntary and electrically evoked leg motion were reduced in pediatric patients with cerebral palsy.

**What is the implication of the main finding?**

- Further optimization of the electrical muscle stimulation protocol, the voluntary motion task, and the MRI acquisition—particularly with regard to scan acceleration—is required.
- These improvements would enable cine phase-contrast MRI to serve as a feasible method for studying muscle dynamics and assessing treatment effects in pediatric patients with neuromuscular diseases.

**Abstract:**

Background/Objectives: Magnetic resonance imaging (MRI) can be used to assess muscle function while performing a motion task within the scanner. Quantitative measures such as contraction velocity and strain can be derived from the images. Cine phase contrast (PC) MRI for time-resolved imaging of muscle function relies on the consistently repeated execution of the motion task for several minutes until data acquisition is complete. This may be difficult for patients with neuromuscular dysfunctions. To date, this approach has been applied only in adults, but not pediatric populations. The aim of this pilot study was to investigate the feasibility of PC MRI for assessing calf muscle function during electrically evoked and voluntary motion in children with cerebral palsy (CP) using open-source hardware and software. Methods: Cine PC MRI was performed at 3T in ambulatory pediatric patients with CP and typically developing children under electrical muscle stimulation (EMS) (*n* = 14/13) and during voluntary plantarflexion (*n* = 4/4) using a home-built pedal with a force sensor. A visual feedback software was developed to enable synchronized imaging of voluntary muscle contractions. Muscle contraction velocity and strain were calculated from the MRI data. Data quality was rated by two readers. Results: During EMS, the velocity data quality was rated as sufficient in 21% of scans in patients compared with 82% of scans in controls. During the voluntary task, all patients demonstrated increased compliance and greater generated force output than during EMS. Voluntary motion imaging was successful in all controls but none of the patients, as motion periodicity in patients was worse during voluntary than during stimulated contraction. Conclusions: Cine phase-contrast MRI combined with EMS or voluntary motion proved challenging in pediatric patients with CP, particularly in those with more severe baseline muscle dysfunction or reduced tolerance to stimulation. In contrast, the approach was successfully implemented in typically developing children. Although the scope of the patient-based findings is limited by data heterogeneity, the method demonstrates considerable potential as a tool for monitoring treatment-related changes in muscle function, particularly in less severely affected patients. Further refinement of the EMS and voluntary motion protocols, together with a reduction in MRI acquisition time, is required to improve motion periodicity, tolerability, and consequently the overall success rate in the intended pediatric patient cohort.

## 1. Introduction

Cerebral palsy (CP) is a sensorimotor dysfunction caused by damage to the developing brain leading to musculoskeletal abnormalities, including weakness and spasticity of the affected muscles [1]. In patients with affected legs, contractures lead to toe-walking (equinus) and gait disorders. Muscles of CP patients may be reduced in volume while respective tendons may be enlarged. It has been proposed that the number of sarcomeres may be reduced in cases of overstretched muscles [2]. Other deformities include hypertonia as well as joint and bone deformities [3,4]. Botulinum toxin A (BTX) has been established as an important treatment modality, particularly for the management of spasticity [5] in CP patients, e.g., to improve gait function by local injections into the calf muscles. In clinics, spasticity and treatment response are assessed with clinical examination (and graded using the Modified Ashworth Scale or Tardieu Scale), electromyography (EMG), and ultrasound (US) elastography (see review [6]). Because they are based on the clinician’s interpretation, clinical examinations using scales are subjective assessments. EMG uses electrodes to measure muscle activity on the surface of the skin and is therefore not well suited to assessing deep muscle activity [6]. US elastography evaluates tissue elasticity during US imaging and has shown changes in muscle elasticity after BTX treatment (see review [7]).

Dynamic imaging of muscle contraction in vivo is feasible with US imaging and MRI. This allows direct assessment of the function of healthy, diseased, and treated muscles. Both methods can be used to derive quantitative measures such as contraction velocity and strain. For example, US has been used to measure these in the arm muscles [8]. US has a higher temporal and spatial resolution but a limited field of view compared to MRI, which is a limitation in large muscles like leg muscles. Time-resolved cine phase contrast (PC) MRI has previously been used to quantify voluntary leg muscle contraction in adults [9,10,11,12,13,14,15,16]; however, its application in children has not yet been reported. The cine PC approach relies on the consistent repetition of the motion task over several minutes until data acquisition is completed. During the acquisition time, complex MRI images consisting of signal magnitude and phase maps are acquired at each time point of the periodic motion cycle with a time resolution of several milliseconds. From these phase maps, velocity vector maps can be calculated showing the motion of the muscle during the cycle. However, the challenge in CP is that patients cannot always voluntarily control their limb movement, and regular periodic motion cannot be performed. Therefore, voluntary exercise paradigms might not be adequate for examination. A PC MRI method for measuring involuntary muscle contraction induced by electrical muscle stimulation (EMS) has been developed [17] and applied in adult healthy volunteers and patients [18,19], but not yet in children. EMS evokes a regular, controlled motion but the stimulation current may feel unpleasant, and the maximum tolerated current may be too low to evoke motion—especially in young children. The aim of this pilot study was to apply time-resolved PC MRI in combination with both EMS and a voluntary paradigm in pediatric patients with CP and healthy children to check and compare the feasibility of both child-adapted experimental open-source setups for assessing calf muscle function. We hypothesized that the EMS setup would generate sufficient PC MRI data quality in CP patients due to the expected periodicity of the evoked motion. The impetus for the initiation of this feasibility study was the potential application of measuring the extent of the paralytic effect of BTX in patients with CP.

## 2. Materials and Methods

### 2.1. Participant Recruitment and Preparation

Two groups of children were recruited between 22 February 2018 and 29 July 2021 and included in the study: 13 healthy, typically developing controls (6 males and 7 females, age range at MRI scan 9 to 16 years, mean age 11 ± 2.7 years, mean height 148 ± 17 cm) and 14 ambulatory patients diagnosed with CP (11 males and 3 females, age range 8 to 15 years, mean age 11 ± 2.1 years, mean height 145 ± 13 cm, 4 bilateral spastic CP and 10 unilateral spastic CP) were prospectively monitored. Participant characteristics can be found in Supplementary Material S1. The participants were enrolled in a clinical study that focused on the effects of BTX treatment on muscle volume and function. Results of the clinical examinations, anatomical MRI scans, and the gait analysis have been presented in [20,21]. The functional MRI experiments were a pilot study within this clinical study. Functional mobility level was classified by the Gross Motor Functional Classification System (GMFCS) I (*n* = 11), II (*n* = 2), and III (*n* = 1) [22]. All patients were toe-walking and were scheduled for BTX injections. Eleven of the 14 CP patients received BTX injections in the affected leg (for dose see Supplementary Material S1), as described in [20]. In the bilaterally affected patients, only the more affected leg (defined as the leg with lower scores in clinical examinations) was treated with BTX and initially scanned. In the healthy controls, the dominant leg (defined as preferred leg for kicking a ball) was scanned once or twice (reason described below). Patients were scanned one to three times (pre-BTX, 6 weeks post-BTX, 12 weeks post BTX); see Table 1 and Table 2.

Patients with CP were recruited from the ambulatory outpatient clinics. Patients considered for BTX therapy and with provided consent from the respective parents were included in the study. Exclusion criteria were generally very incompliant and agitated patients not able to lie still in the MRI scanner for a longer period of time, additional severe pathologies, general contraindications for an MRI, previous surgeries on the affected limb(s), claustrophobia, difficulties in following instructions in the scanner, and lacking consent. Healthy controls were recruited by asking the families of outpatient clinic patients and medical center staff. Only children without severe health problems and without muscle-associated diseases were included. The other inclusion/exclusion criteria were the same as for the patients. The study was conducted in accordance with the Declaration of Helsinki. Prior to participation, informed written consent was obtained from the participants’ parents and additionally from those participants aged 12 years or older. The study was approved by the local ethics committee (Ethikkommission Nordwest- und Zentralschweiz, EKNZ BASEC 2016-01408).

Three patients dropped out of the study after the first time point (two of them were not eligible for BTX treatment based on clinical examination and gait analysis, one of them could not participate in the follow-up scans due to COVID restrictions). One patient could not be scanned on the third time point due to scanner downtime.

**Figure 1:**
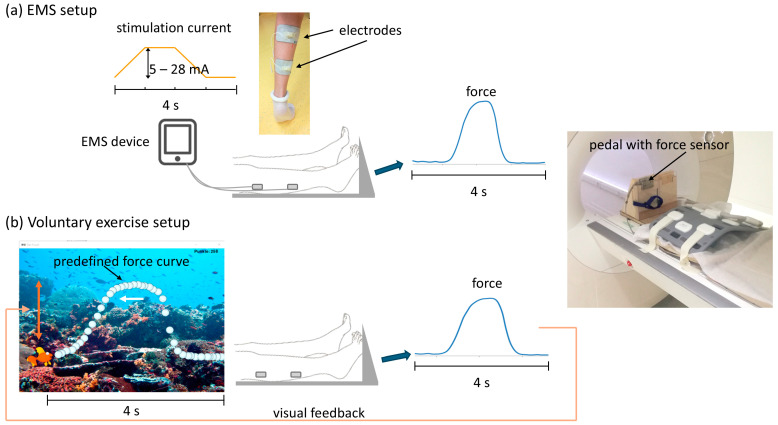
Setup for the EMS (**a**) and voluntary exercise (**b**) experiments. (**a**) The EMS device had a 4 s stimulation cycle. The self-adhesive electrodes were placed on the calf of the participants (as shown for a toe-walking patient in the photo). On the right, the home-built pedal with the force sensor is shown which was placed on the patient table. (**b**) For voluntary exercise, a predefined periodic force curve with a period of 4 s was projected on a screen in the scanner room (screenshot on the left). The participant moved the fish up and down by pressing the pedal and was supposed to hit as many bubbles as possible (moving from right to left) of the predefined curve.

The triceps surae muscle group of the (more) affected leg (the dominant leg in healthy controls) was imaged in a 3T whole body clinical MRI scanner (Prisma, Siemens Healthineers, Erlangen, Germany). Two self-adhesive electrodes with MRI-visible glycerine capsules as markers taped to the surface were attached to the muscle belly (see below and Figure 1a). The distance of the markers from the popliteal fossa was noted and the position was photographed to ensure that the electrodes (and the imaging slices according to the markers) could be placed in the same position for subsequent sessions. The children were placed feet first supine on the scanner table, and a flexible 18-channel body coil (Siemens Healthineers, Erlangen, Germany) was placed on the lower legs. The foot was attached with straps to a home-built foot pedal device with a built-in open-source force sensor (Figure 1a) [23]. Before starting the scans, the children were asked to press their forefoot on the pedal with maximum force. The maximum voluntary force (MVF) was measured as the mean of two trials. One to three PC MRI experiments (evoked motion—using up to two PC MRI protocols with different velocity encoding VENC—and voluntary motion, see below) with periodic plantarflexion were performed in one session in each participant, as listed in Table 1 and Table 2. The number of experiments during a scan session was dependent on the compliance of the participants. In the course of the study, we added a sequence with VENC 10 cm/s to the protocol because we found that an increased sensitivity was needed compared to VENC 25 cm/s (see Section 4). The voluntary paradigm was performed in four patients (in two of these patients at two time points) and in four healthy controls (Table 1 and Table 2). The voluntary paradigm was added in the course of the study, so that it was performed in all patients and controls whose MRI scans were scheduled after June 2020. In three of these controls, this scan session took place three years after the first MRI scan with evoked motion only, so that the evoked motion protocol with VENC 10 cm/s was also repeated at the second MRI session. Finally, in eight patients the evoked motion was also measured in the contralateral, non-treated leg six weeks post BTX-injection.

### 2.2. EMS Setup

**Figure 2:**
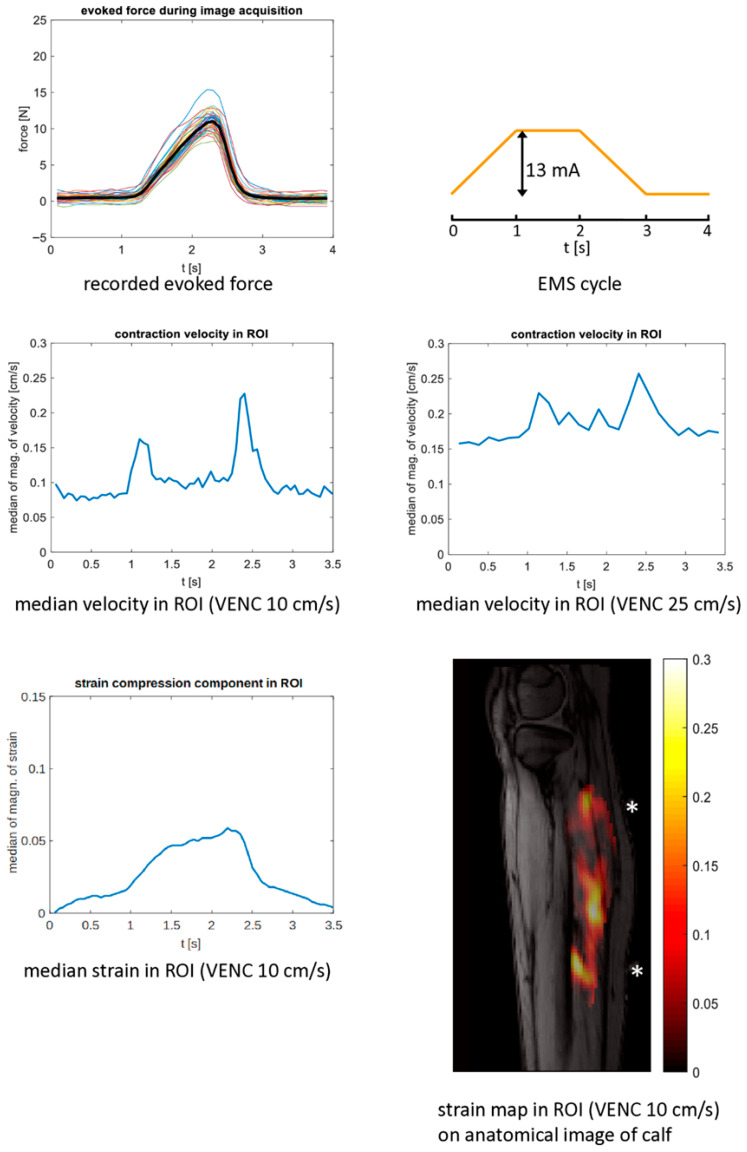
Cine phase contrast MR experiment with synchronized EMS in a 14-year-old girl with bilateral cerebral palsy. The experiments were performed in the more affected leg before BTX treatment. **Top left**: time courses of the evoked force on the pedal with the mean force curve (over the whole experiment) in black. **Top right**: current amplitude of the EMS cycle. **Middle left**: time course of the velocity magnitude (ROI median) showing two peaks, one at contraction (left peak) and one at release (right peak) acquired with VENC 10 cm/s. The MR acquisition window (and therefore the velocity time scale) was 3.5 s during each EMS-cycle of 4 s. **Middle right**: for comparison, time course of the velocity magnitude (ROI median) acquired with VENC 25 cm/s. **Bottom left**: time course of the strain magnitude (ROI median of the strain compression component, acquired with VENC 10 cm/s). **Bottom right**: strain map at the temporal phase when the maximum occurs overlaid on an anatomical image of the calf (magnitude of the strain compression component, VENC 10 cm/s). The positions of the electrodes for stimulation are marked with asterisks. EMS: electrical muscle stimulation, VENC: velocity encoding.

The setup for the EMS scans was similar to that described in [17]. To induce a periodic contraction, a low-cost two-channel commercially available EMS device (InTENSity Twin Stim III TENS and EMS Combo, Current Solutions LLC, Austin, TX, USA; intended for EMS muscle training at home) was used. The device was connected to 5.1 × 8.9 cm^2^ rectangular self-adhesive gel-based electrodes (TENSUnits.com, Largo, FL, USA) placed over the gastrocnemii (approx. 7 cm below the popliteal fossa) and soleus (approx. 10 cm below the first electrode) muscles (Figure 1a). The EMS cycle (1 s ramp up, 1 s plateau, 1 s ramp down, 1 s recovery, Figure 1a left and Figure 2 top right) had 30 pulses/s and a pulse width of 300 µs. The evoked force was recorded during the experiment with the force sensor in the pedal. Before starting the scan, the amplitude of the stimulation current was raised within the comfort levels (healthy controls 13–28 mA, patients 5–20 mA) to a point where an induced visible force output of at least 5% MVF was achieved, unless if the child felt uncomfortable then the stimulation current was reduced. Two three-directional cine gradient echo PC velocity encoding sequences were applied using the same setup. To synchronize the sequences to the EMS cycle, the second channel of the EMS device was used to generate a trigger signal at the start of each stimulation cycle. The acquisition window was 3.5 s during each EMS-cycle of 4 s. The sequence with VENC 10 cm/s was acquired with following parameters: one parasagittal slice based on the position of the two markers, voxels: 2.2 × 2.2 × 5.0 mm^3^, echo time 9.7 ms, bandwidth/pixel 400 Hz/px, flip angle 10°, FOV 280 × 140 mm^2^, acquisition time 2:28 min, 67 temporal phases, and temporal resolution of 52 ms. The sequence with VENC 25 cm/s was performed with the following parameters: three parasagittal slices (the central slice placed on the position of the two markers) with a spatial resolution of 2.2 × 2.2 × 5.0 mm^3^, echo time 7.2 ms, bandwidth/pixel = 400 Hz/px, flip angle 10°, FOV 280 × 140 mm^2^, three k-space lines per segment, acquisition time 3 min, 27 temporal phases, and temporal resolution of 126 ms.

### 2.3. Voluntary Exercise Setup

**Figure 3:**
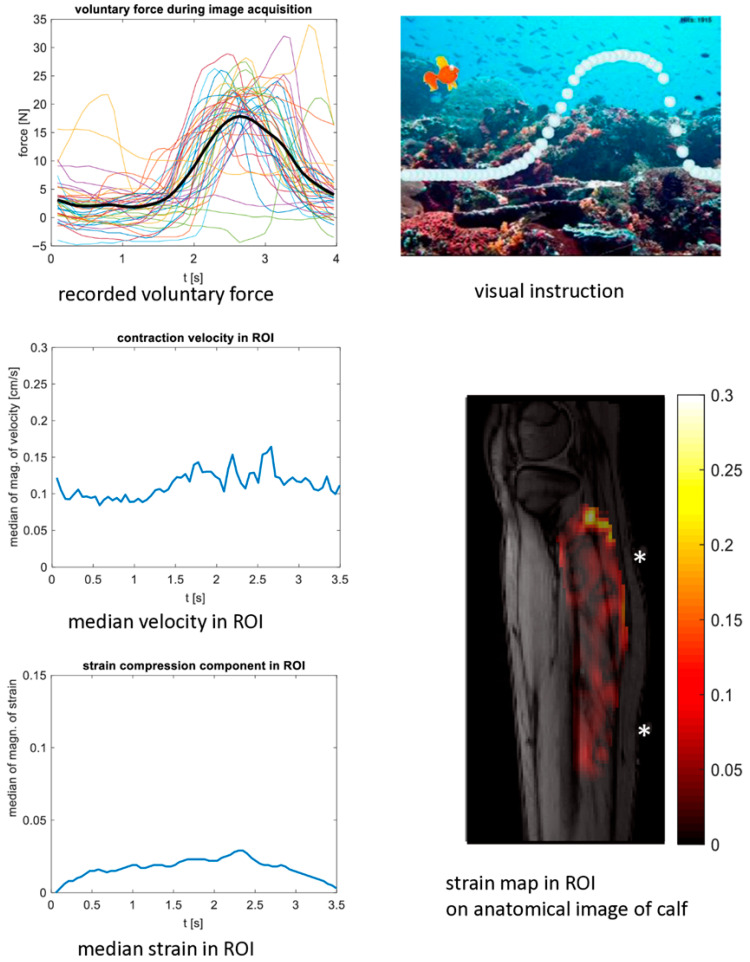
Cine phase contrast MR experiment with voluntary motion in a 14-year-old girl with bilateral cerebral palsy. The experiments were performed in the more affected leg before BTX treatment. The patient is the same as in Figure 2. **Top left**: time courses of the force on the pedal with the mean force curve in black. **Top right**: screenshot of the visual paradigm for instruction and feedback for the participant. **Middle left**: time course of the velocity magnitude (ROI median, acquired with VENC 10 cm/s) showing several small peaks. The MR acquisition window (and therefore the velocity time scale) was 3.5 s during each voluntary paradigm period of 4 s. **Bottom left**: time course of the strain magnitude (ROI median of the strain compression component, acquired with VENC 10 cm/s). **Bottom right**: strain map at the temporal phase when the maximum occurs overlaid on an anatomical image of the calf (magnitude of the strain compression component). The positions of the electrodes for stimulation are marked with asterisks. VENC: velocity encoding. BTX: botulinum toxin A.

In a subgroup of patients and healthy controls, a voluntary exercise paradigm was projected on a screen in the scanner room after a short resting period following the scans with evoked motion. The paradigm consisted of a periodic curve with a pre-set amplitude (depending on the individual MVF, range 10–20 N in patients, 30–40 N in healthy children) and a period of 4 s. The shape of the predefined curve was the average of the force time courses of two adult volunteers who had previously performed a periodic exercise (pressing the pedal for 1 s after a trigger signal every 4 s) in the same setup. The force generated by the plantarflexion on the pedal was recorded and overlaid in real time on the predefined curve. The paradigm was presented in the form of a video game: the fish—the force on the pedal—was supposed to hit as many bubbles—the predefined curve—as possible; see Figure 1b and Figure 3, top left. The patient was supposed to follow the predefined curve by pressing the pedal. After a training period of 5 min the voluntary motion was performed during the scan. The code for the display of the exercise paradigm and the real-time force values was written in Python 3 (code available on https://github.com/BAMMri, accessed on 10 January 2026). The cine PC sequence with VENC 10 cm/s was applied with the acquisition synchronized to the default periodic curve, i.e., the acquisition window (3.5 s) started every 4 s in the resting phase. The sequence with VENC 25 cm/s was not performed for the voluntary exercise paradigm (due to its lower sensitivity compared to VENC 10 cm/s, see Section 4).

### 2.4. Data Processing

Data were processed offline using Matlab (The Mathworks, Inc., Natick, MA, USA, release R2017a). The Matlab code and Python 3 versions are available on https://github.com/BAMMri (accessed on 10 January 2026). To visualize the contraction time course, a ROI was manually drawn on the triceps surae muscles visible in the image slice and the velocity vector field was displayed for each temporal phase [17]. For the VENC 25 cm/s dataset, a ROI was drawn on the slice, which showed the largest area of triceps surae muscles. For the velocity time course, the ROI median of the magnitude of the velocity vectors was calculated for each phase. Strain vectors were extracted from the velocity and displacement fields as described in [17]. The tensors were diagonalized, and the principal eigenvalue with the largest absolute value, representing the compression component, was visualized. For the strain time course, the ROI median of the magnitude of the strain compression component was calculated for each temporal phase. Data from control 3 were not processed as the MRI scan and force measurement under stimulation failed due to severe motion artifacts.

The velocity time courses were used to assess data quality. Peaks in the velocity time courses were identified by two readers by visual inspection. Data quality was rated as good and the PC MRI scan was rated as successful when two clearly identifiable velocity peaks were observed by both readers: one corresponding to muscle contraction and, approximately one second later, a second peak corresponding to muscle relaxation. In both cases, the peak amplitudes exceeded the baseline noise in the plot of the velocity time course. Visibility of only one peak or several not well-defined peaks was graded as mediocre, no clearly identifiable peaks were graded as not sufficient. If the data quality was sufficient, maximum strain values and strain maps were calculated as desired outcome parameters as described in [17,24]. The maximum median velocity in the ROI was calculated as the difference between the maximum and minimum values in the velocity time course. The maximum median strain in the ROI was determined as the maximum value in the strain time course.

Mean force time courses over the 4 s period were calculated from the recorded force data during each cine scan (i.e., as recorded directly with the foot pedal). The maximum force during stimulation was calculated as the difference between minimum and maximum values of the mean force time course. The relative maximum force was calculated as the percentage of the MFV for each participant. If there was no detectable periodic motion in the force data, the maximum force was set to zero.

## 3. Results

**Figure 4:**
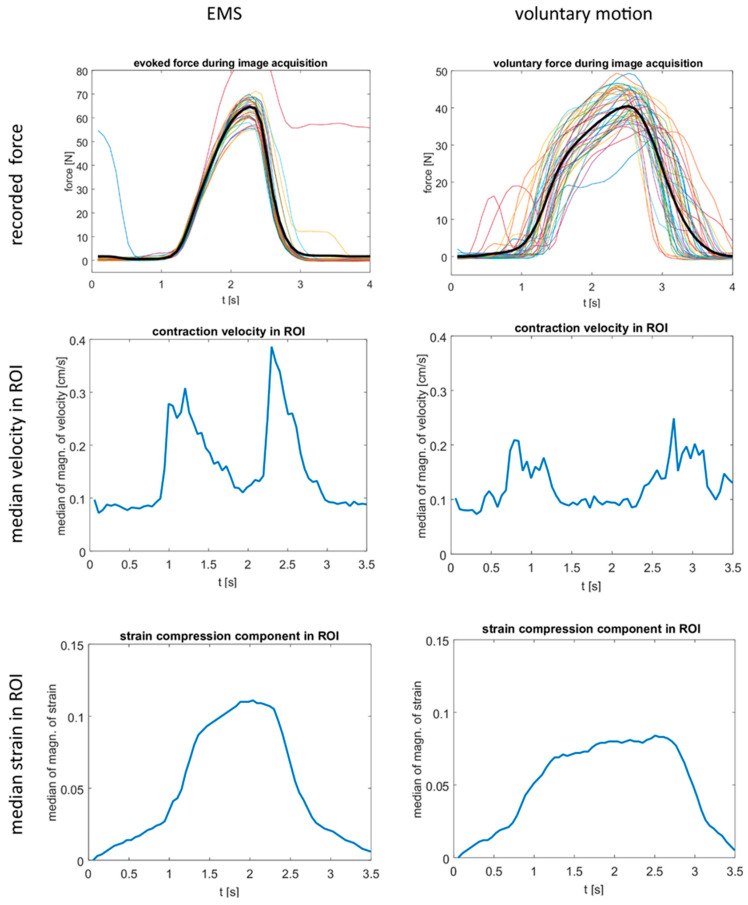
Cine phase contrast MR experiment with synchronized EMS and voluntary motion in a 9-year-old typically developing girl. The left column shows the results during stimulated motion (EMS), the right column during voluntary motion. **Top row**: time courses of the force on the pedal with the mean force curve in black for the stimulated and voluntary motion, respectively. **Middle row**: time course of the velocity magnitude (ROI median, acquired with VENC 10 cm/s) showing two peaks at contraction (left peak) and release (right peak). The peaks for the voluntary motion were relatively broad and not so well defined, corresponding to the lower periodicity of the force time courses. **Bottom row**: time courses of the strain compression component (ROI median, acquired with VENC 10 cm/s). EMS: electrical muscle stimulation, VENC: velocity encoding.

**Figure 5:**
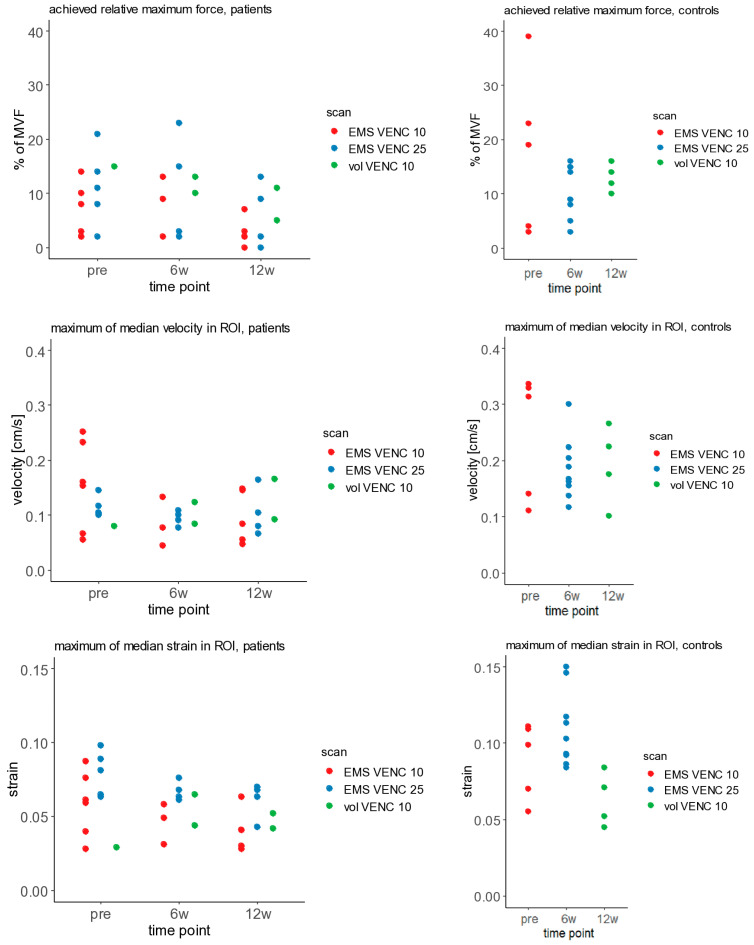
Overview of quantitative parameters derived from scans with sufficient data quality in patients with cerebral palsy (**left**, in the treated leg) and controls (**right**). **Top row**: relative force on the pedal in percentage of maximum voluntary force (MVF). **Middle row**: maximum velocity in the time courses (ROI median). **Bottom row**: maximum strain in the time courses (ROI median). Red dots represent scans with EMS acquired with VENC 10 cm/s, blue dots represent scans with EMS acquired with VENC 25 cm/s, green dots represent scans during voluntary exercise acquired with VENC 10 cm/s. The patients were scanned on up to three time points: pre, 6 weeks post, and 12 weeks post botulinum toxin A injection.

The maximum tolerated stimulation current and the force developed at this current varied considerably (Table 1 and Table 2), even within the same participant scanned in two sessions. The time course of the contraction speed showed two distinct peaks for muscle contraction and release (as shown in Figure 2 for patient 14) in 15 of 72 scans in patients and 14 of 17 scans in healthy children (as shown in Figure 4 for control 13). In these cases, the data quality was rated as good (Table 1 and Table 2), which was sufficient to calculate strain maps from the velocity vector. In these cases, the strain time courses showed a bell-shaped curve (Figure 2 and Figure 4). However, the strain maps (compression components) for the temporal phase where the maximum strain occurs were inhomogeneous with pronounced local differences (Figure 2 and Figure 3). The maximum median velocities and strains and the maximum achieved relative force values are displayed in Figure 5. The maximum strain value in the time courses was in the range of 0.063 to 0.089 for the *n* = 5 patient experiments with mediocre or good data quality pre BTX (acquired with VENC 25 cm/s), resulting in a mean of 0.079 and a median of 0.081. In the typically developing children, the corresponding range was 0.084 to 0.146 (*n* = 9, acquired with VENC 25 cm/s), resulting in a larger mean and median of 0.11 and 0.10, respectively. All the time courses for contraction/release velocity, strain, and evoked force during EMS can be found in Supplementary Material S2, and all the time courses for the direct comparison between EMS and voluntary motion paradigm can be found in Supplementary Material S3. MVF values are provided in Supplementary Material S4. As expected, MVF was generally smaller in the affected/treated legs compared to the non-affected/non-treated legs in patients, as well as compared to the legs of controls. There was no clear difference in data quality of the treated leg compared to the non-treated leg at 6 w post BTX.

Contraction and release velocities (ROI medians) were considerably below 1 cm/s. Due to the low contraction speeds, the sequence with the reduced VENC of 10 cm/s, i.e., with the increased sensitivity for lower velocities, produced a higher signal-to-noise ratio, as can be seen in the velocity time courses (Supplementary Material S2). An increased noise level led to a larger offset in the plots with VENC 25 cm/s vs. 10 cm/s since the velocity time courses were calculated with absolute values. The data quality (defined by the three ratings “good,” “mediocre”, and “not sufficient”) of VENC 25 cm/s and VENC 10 cm/s was directly compared in 32 cases where scans with both VENCs were acquired in one session. In seven cases, the data quality was rated higher for VENC 10 cm/s, for the rest of the cases the data quality rating did not change between the two different VENC settings.

Regarding the voluntary motion experiments, the pre-set amplitude of the voluntary force paradigm could be generated by the participants without much effort. The amplitude of the voluntary force was larger than the amplitude of the stimulated force for all patients (Table 1 and Table 2, Supplementary Material S3). However, the periodicity was worse, especially in patients (as shown in Figure 3 for patient 14; plots for all are shown in Supplementary Material S3). It was possible to resolve two distinct peaks in four out of four scans in healthy children (i.e., data quality was rated as good, as shown in Figure 4 for control 13 and in Supplementary Material S3), and in none out of six scans in patients (i.e., data quality was rated as mediocre—as in Figure 3—or not sufficient; see Table 1).

## 4. Discussion

To date, no studies have examined leg muscles in children using time-resolved functional MRI methods. This includes both voluntary and stimulated motion, as well as typically developing children and pediatric patients. The aim of this pilot study was to find an experimental open-source setup that would allow us to assess muscle function in pediatric patients with neuromuscular diseases which could be used in future studies, e.g., in CP patients to evaluate the spatial extent of the paralytic effect of BTX in strain maps. The cine PC approach relies on the consistent repetition of a movement task over several minutes, which is problematic in patients with poor coordination and weakness, such as in CP patients. Periodic motion can be achieved with the EMS paradigm. If the tolerated stimulation current is insufficient to evoke a detectable periodic motion (i.e., a detectable periodic force), then the dynamic MR measurement fails. In our study, this occurred in approximately 60% of the sessions with pediatric patients, where no velocity peaks were visible in the velocity time courses (data quality rated as not sufficient). The reduced success rate in patients due to the limited force generation and the resulting velocities falling below the detection threshold, likely due to the underlying pathology. MVF (Supplementary Material S4) in the (more) affected leg was smaller than in controls, which means that the force capacity of the affected muscle was generally reduced, also during EMS. Successful treatment with BTX leads to paralysis of the injected parts of the muscle, thereby further reducing the force and motion capacity. However, the success rate was also reduced in the unaffected/less affected leg (non-treated), despite larger MVF. This suggests that an additional limiting factor in patients is their reduced tolerance to stimulation current compared to healthy controls. Accordingly, the stimulation currents tolerated during the experiments were lower in patients than in controls (Table 1 and Table 2). A certain level of stimulation current was required to evoke motion with sufficient force, which was not achieved in patients within their comfort range. Reduced stimulation currents resulted in decreased velocities [17] and lower force outputs which impedes sufficient assessment of muscle function.

For comparison, we developed a setup with an open-source visual feedback software for a voluntary motion paradigm. It was well tolerated in both CP patients and healthy controls, and the children were highly motivated. The force output during voluntary motion was larger than that achieved with the maximum tolerated stimulation current, which was promising for detectable contraction velocities. The periodicity of the voluntary motion over the acquisition time was sufficient in controls to resolve two distinct velocity peaks for muscle contraction and release. However, this was not the case with patients. The recordings of the force on the pedal clearly showed the lack of periodicity in the voluntary motion. The force curves acquired during stimulation also revealed extra voluntary movements (see Supplementary Materials S2 and S3). Thus, these recordings were very helpful in explaining the data quality of the cine PC MRI scans, which depended on the achieved force and the resulting detectable contraction velocities, as well as on the periodicity of the motion over the scan time. It was also very practical to be able to adjust the stimulation current with the participant already in the correct exam position inside the scanner using the force curves. In principle, the force may be used to achieve comparability between scans. The repeatability study in adults by our group [18] showed that strain values obtained from EMS-evoked muscle contractions were highly repeatable (using statistical methods including intraclass correlation coefficients and repeatability coefficients) and strain maps were qualitatively similar when the evoked force during the scan was used as a reference for standardization. Based on the results of this repeatability study, we selected strain and strain maps as outcome parameters and recorded the achieved force. Another potential outcome parameter in cine PC MRI is the maximum velocity in a ROI: S. Sinha et al. showed that it decreased during the course of muscle atrophy in the lower leg [25]. Strain rate (which can also be derived from cine PC MRI data) is also a promising outcome parameter: U. Sinha et al. measured smaller strain rate values in elderly compared to young adults at similar force levels during plantarflexion [11,12].

Comparing EMS and voluntary motion, the evoked strain seemed to be larger than the strain during voluntary motion in both patients and controls. Overall, the strain appeared to be smaller in the patient group than in the control group. The strain maps were relatively inhomogeneous in the contracting muscles, thus no convincing conclusions about regional differences in the strain could be drawn. Due to the large number of experiments with insufficient data quality, it was not possible to draw definite conclusions regarding strain quantification. In adults, the compliance of EMS and the tolerated stimulation current was higher than in children, as demonstrated in [17,18,19]. In these studies, two distinct velocity peaks were detected, and the data quality was good enough to generate strain maps. The observed values for the maximum strain in this study with children (in experiments with good or mediocre data quality) were in the same range as the strain values for adults in the calf during stimulated plantarflexion [18]. The peak contraction values for evoked motion were typically higher in adults, ranging from 0.5 to 10 cm/s in the thigh. Consequently, VENC values of 25 cm/s (thigh) and 30 cm/s (lower leg) were used [17,18,19]. An inhomogeneous pattern in the strain maps as in our study has also been observed in healthy adults in experiments with mediocre data quality (i.e., no identifiable two velocity peaks present) [17,18].

In our study, worse and more heterogeneous results in the patient group compared to the controls were expected due to the pathology itself. Some muscle dynamics, however, could be observed with our cine PC MRI protocol in some of the patients, probably reflecting less severely affected or more task-cooperative patients. Despite all these challenges, our data suggest that voluntary motion-based and EMS-driven MR-protocols could be used for muscle assessment in future studies, especially in less severely affected patients. Voluntary tasks resulted in greater force generation and increased compliance, whereas better motion periodicity was achieved with EMS. With an increased success rate, cine PC MRI could serve as a marker for follow-up assessment of muscle function in less severely affected or more compliant patients. It may become a diagnostic adjunct, in addition to already existing methods, e.g., gait assessment and US, for objective evaluation of muscle function in the context of BTX treatment in CP patients. This is important as cine PC MRI has some advantages over US and gait analysis, as it can objectively and quantitatively assess a wider extent of muscles, including deeper muscle groups with higher resolution. However, for a realistic application in the future, the protocols for EMS, voluntary task, and cine PC MRI need to be further adapted to increase tolerability and success rate in pediatric patients.

One way to increase the success rates for these kinds of acquisitions may be a reduction in scan time. Compressed sensing reconstruction has already demonstrated the ability to speed up CP contrast MRI in the lower leg down to 40 s for one slice [26] and 1 min 18 s for three slices [27]. Patients may be able to achieve sufficient periodicity in the voluntary motion over such a short time period. Furthermore, compressed sensing can also enable three-dimensional (3D) coverage of the entire muscle belly in a reasonable acquisition time [9,10]. A longer training period than the five minutes used in our study could also help to increase the periodicity and therefore improve data quality. However, an increase in the duration of the plantarflexion exercise may lead to fatigue of the patients and irregular motion.

The critical point in the EMS experiments remains to increase acceptance and improve the sensation of the stimulation. Different settings of the stimulation parameters may be better tolerated. We chose our settings according to our experience in adults and used stimulation parameters similar to the settings for EMS used in physiotherapy for CP patients [28]. However, in our experience, the maximum tolerated current varied from session to session in the same participant and depended on the mood and physical condition of the child on the day of the exam. We kept the stimulation frequency and pulse width values fixed for all participants and determined the stimulation current in a procedure similar to the experiments in adults, but aimed for a decreased force of 5% of MVF compared to 15% in adults [18]. There was no clear trend indicating that the method works better in older children or in children with higher body mass index (BMI). Compliance depends on individual tolerance to sensation evoked by EMS [29]. This can be achieved either by adjusting the EMS parameters for each participant or by longer and more frequent training periods (to get used to the sensation). In healthy controls, the short session to adjust the stimulation current seemed to be sufficient training and the EMS parameters were fine in most participants. This was different in patients. More training sessions with a wider range of protocol adjustments may be necessary. Increasing the EMS pulse width while reducing the current or changing the frequency can increase the tolerance to achieve the best possible muscle contraction [29]. Additionally, it was very important for the success of the MRI acquisition to provide a pleasant atmosphere and to entertain the children by showing a movie or by other distractions during the exam inside the scanner.

An alternative method to track motion with MRI is spin tagging which has also been performed in leg muscles [25,30]. Tissue magnetization is saturated in a spatial pattern of thin bands. These tagged bands of reduced signal intensity allow the visualization of the tissue motion in subsequent images. The advantage of the spin-tagging method is that only a few motion cycles are required to obtain a data set, thus avoiding fatigue and loss of periodicity, which is a problem with the many motion cycles in the cine PC method. The disadvantage is the more complex post-processing to obtain displacement and strain maps.

One limitation of this pilot study was the small number of patients and healthy controls. Additionally, the groups were heterogeneous in terms of age. More participants are necessary to investigate the influence of age, BMI, and MVF on the performance of the methods. The data quality was assessed by visually inspecting the velocity time courses, which introduced subjectivity and potential inter-reader variability. This may have influenced the subsequent inclusion or exclusion of scans in further data analysis. A further limitation of this study was that the voluntary paradigm was only applied to a subgroup of participants. This decision was based on our assumption that CP patients would not be able to perform a periodic voluntary motion for several minutes; therefore, only a limited number of children were asked to perform this task for comparison. The test in this subgroup of patients confirmed these doubts. Another aspect that limited the study was the inherent inhomogeneity in the VENC values of the study. Since it was determined after the initial tests and the start of the study that a low VENC was more appropriate for children, a delayed decision was made to add the sequence with VENC 10 cm/s. All the scans with the voluntary motion (which took place in the third year of the study) were acquired with the VENC 10 cm/s sequence only. Lower VENC offered an increased sensitivity, but only one slice could be acquired in two and a half minutes compared to three slices in three minutes with the VENC 25 cm/s sequence. To achieve a lower VENC a higher gradient strength or longer gradient duration is required which changes the timing of the sequence. Thus, multislice acquisition with a low VENC in a short acquisition time was not feasible. However, the timing of the single-slice sequence with lower VENC allowed a better temporal resolution.

The repeatability of the method was not demonstrated in this feasibility study. Test–retest measurements need to be performed in children, as has been done in adults [18], before treatment effects can be properly evaluated in a clinical trial. Being a methodological and feasibility study, the primary aim of this study was not to investigate the treatment effect with PC MRI. Since the experiments were performed in patients enrolled in a BTX-treatment study where anatomical MRI scans were performed [20], we took the opportunity to additionally acquire PC MRI data in the same patient pool at multiple time points. However, it was not possible to achieve sufficient data quality pre and post BTX in the same patient. As a result, a comparison of strain maps pre and post BTX to locate paralyzed parts of the muscle was not possible.

## 5. Conclusions

In conclusion, this feasibility study showed that cine PC MRI in combination with EMS or a voluntary motion paradigm to assess plantarflexion was challenging in pediatric CP patients, whereas it worked well in typically developing children. EMS makes repeatable exercise in the scanner potentially more feasible since an external device controls the movement. However, with the current EMS protocol, we could not demonstrate the feasibility in the intended pediatric patient cohort, which is an important finding of the study. Therefore, further research is needed to adapt the EMS protocol and investigate the potential benefit of additional EMS training sessions on EMS tolerability and thus on compliance and success rate. The voluntary motion paradigm with visual feedback is a child-friendly approach, but also, further studies with this setup are needed that investigate if faster MRI acquisition and longer training time may improve periodicity. The feasibility and applicability of both setups are still limited at this stage for being implemented in the clinical routine for treatment follow-up assessment. After successful adaptations, cine PC MRI can become an applicable tool to study muscle dynamics before and after treatment in pediatric patients with neuromuscular diseases.

## Figures and Tables

**Table 1:** Overview of performed scans and data quality in patients *.

	EMS								Voluntary Motion		
Patient Number	pre BTX, VEN25	pre BTX, VEN10	6w post BTX, VENC 25	6w post BTX, VENC 10	6w post BTX, non-treated leg, VENC 25	6w post BTX, non-treated leg, VENC 10	12w post BTX, VENC 25	12w post BTX, VENC 10	pre BTX, VENC 10	6w post BTX, VENC 10	12w post BTX, VENC 10
1	15 mA		14 mA	14 mA	13 mA	13 mA	18 mA	18 mA			
	5%		4%	1%	5%	0%	3%	2%			
2	16 mA		15 mA	15 mA	15 mA	15 mA	16 mA	16 mA			
	21%		9%	9%	3%	2%	2%	2%			
3	16 mA		15 mA		17 mA		19 mA	19 mA			
	2%		3%		1%		13%	3%			
4	15 mA	15 mA	18 mA		19 mA		19 mA	19 mA			
	14%	14%	23%		1%		9%	7%			
5	14 mA	14 mA	9 mA	9 mA	7 mA	7 mA	16 mA	16 mA			
	10%	10%	15%	13%	16%	15%	2%	2%			
6	8 mA	8 mA	15 mA	15 mA			8 mA	10 mA			
	2%	2%	3%	1%			0%	3%			
7	10 mA	20 mA									
	3%	2%									
8	10 mA										
	1%										
9	14 mA	14 mA	15 mA	15 mA	16 mA	18 mA	13 mA	13 mA			10 N
	8%	8%	1%	0%	0%	0%	2%	2%			5%
10	13 mA	14 mA									
	2%	3%									
11	6 mA	7 mA									
	0%	0%									
12	5 mA		7 mA	7 mA	5 mA	5 mA	10 mA	10 mA			20 N
	0%		0%	0%	0%	0%	0%	0%			11%
13	12 mA		19 mA	19 mA			12 mA	14 mA		20 N	20 N
	0%		2%	1%			0%	0%		10%	21%
14	13 mA	13 mA	8 mA	8 mA	8 mA	8 mA			20 N	20 N	
	11%	10%	0%	1%	0%	0%			15%	33%	

* The phase contrast cine scans were performed pre and post botulinum toxin A (BTX) injection with VENC values 10 or 25 cm/s and during stimulated motion (EMS) or a voluntary exercise task. Data quality was rated using the plots of the velocity time courses as follows: Two clearly identifiable velocity peaks were graded as good (blue cells). Visibility of only one peak or several poorly defined peaks was graded as mediocre (yellow cells); no clearly identifiable peaks were graded as not sufficient (red cells). Empty cells mean the scans were not performed. The scans were performed in the treated leg. At 6w post BTX also the non-treated leg was scanned in some patients. The amplitude of the stimulation current (in mA) or the pre-set force level for the voluntary paradigm (in N) is also stated along with the achieved relative maximum force (in %). VENC: velocity encoding.

**Table 2:** Overview of performed scans and data quality in controls (healthy, typically developing children) *.

	EMS			Voluntary Motion	
Control Number	1st Session, VENC 25	1st Session, VENC 10	2nd Session, VENC 10	1st Session, VENC 10	2nd Session, VENC 10
1	20 mA, 1%		25 mA, 4%		30 N, 10%
2	23 mA, 1%				
3	24 mA, 0%				
4	26 mA, 15%				
5	28 mA, 9%				
6	22 mA, 8%				
7	20 mA, 14%		23 mA, 23%		40 N, 14%
8	20 mA, 9%		26 mA, 39%		40 N, 16%
9	16 mA, 15%				
10	23 mA, 5%				
11	21 mA, 16%				
12	13 mA, 3%	13 mA, 3%			
13		22 mA, 19%		40 N, 12%	

* The phase contrast cine scans were performed in the dominant leg with velocity encoding (VENC) values 10 or 25 cm/s and during stimulated motion (EMS) or a voluntary exercise task. Data quality was rated using the plots of the velocity time courses as follows: Two clearly identifiable velocity peaks were graded as good (blue cells). No clearly identifiable peaks were graded as not sufficient (red cells). Empty cells mean the scans were not performed. The amplitude of the stimulation current (in mA) or the pre-set force level for the voluntary paradigm (in N) is also stated along with the achieved relative maximum force (in %).

## Data Availability

The relevant processed data are described and shown within the manuscript and its Supplementary Material files. The medical imaging data that support the findings of this study are not openly available due to reasons of sensitivity and are available from the corresponding author upon request.

## Supplementary Materials

The following supporting information can be downloaded at: https://www.mdpi.com/article/10.3390/children13010116/s1. Supplementary Material S1: Participant characteristics of CP patients and controls; Supplementary Material S2: Overview of all acquired time courses of the experiments under EMS, Supplementary Material S3: Comparison of the results under EMS and voluntary motion; Supplementary Material S4: Maximum voluntary force for plantarflexion in pediatric CP patients and healthy, typically developing children (controls).

## Abbreviations

The following abbreviations are used in this manuscript:

PC	Phase contrast
CP	Cerebral palsy
MRI	Magnetic Resonance Imaging
EMS	Electrical muscle stimulation
BTX	Botulinum toxin A
EMG	Electromyography
US	Ultrasound
GMFCS	Gross Motor Functional Classification System
VENC	Velocity encoding
MVF	Maximum voluntary force
FOV	Field of view
ROI	Region of interest
BMI	Body mass index
