# Supplementary material for: Cine Phase Contrast Magnetic Resonance Imaging of Calf Muscle Contraction in Pediatric Patients with Cerebral Palsy and Healthy Children: Comparison of Voluntary Motion and Electrically Evoked Motion
Source: Children (Basel). 2026 Jan 13;13(1):116. doi: 10.3390/children13010116 (PMC12839631; doi:10.3390/children13010116)
Supplement: Supplementary file 1 [file children-13-00116-s001.zip › S1.pdf]

Participant characteristics of CP patients

patient number	sex	age	type of CP	GMFCS	height range [cm]	BMI percentile range [%]	BTX total dose [U]	treated muscles
1	m	12	uni	I	140-150	0-10	150	Gl+Gm+S
2	m	11	uni	I	150-160	90-100	150	Gl+Gm+S
3	f	11	uni	I	140-150	20-30	150	Gl+Gm+S
4	m	11	bi	I	130-140	40-50	150	Gl+Gm+S
5	m	12	bi	II	130-140	0-10	225	Gl+Gm+S
6	m	9	bi	III	120-130	0-10	200	Gl+Gm
7	m	10	uni	II	150-160	70-80	0	_
8	m	15	uni	I	160-170	60-70	0	_
9	m	9	uni	I	130-140	90-100	250	Gl+Gm+S
10	m	8	uni	I	130-140	0-10	0	_
11	f	11	uni	I	140-150	10-20	200	Gl+Gm+S
12	m	8	uni	I	120-130	50-60	150	Gl+Gm
13	m	12	uni	I	150-160	0-10	100	Gl+Gm
14	f	14	bi	I	160-170	50-60	300	Gl+Gm+S

11 of the 14 patients were treated with botulinum toxin A (BTX) injection in the calf muscles (gastrocnemius lateralis Gl, gastrocnemius medialis Gm, soleus S) of the (more) affected leg. CP: cerebral palsy, m: male, f: female, uni: unilateral, bi: bilateral, GMFCS: Gross Motor Functions Classification System, BMI: Body Mass Index, U: Units

Participant characteristics of controls (healthy, typically developing children)

control number	sex	age (1st session)	height range (1st session) [cm]	BMI percentile range (1st session) [%]	age (2nd session)	height range (2nd session) [cm]	BMI percentile range [%]
1	m	9	130-140	60-70	12	140-150	50-60
2	m	12	160-170	60-70			
3	f	10	140-150	50-60			
4	m	9	130-140	30-40			
5	f	16	170-180	0-10			
6	f	16	170-180	50-60			
7	f	9	130-140	30-40	12	150-160	30-40
8	f	9	130-140	0-10	12	150-160	0-10
9	m	13	160-170	0-10			
10	m	9	120-130	20-30			
11	f	14	160-170	20-30			
12	m	10	130-140	40-50			
13	f	9	140-150	10-20			

m: male, f: female, BMI: Body Mass Index
