# Supplementary material for: Cine Phase Contrast Magnetic Resonance Imaging of Calf Muscle Contraction in Pediatric Patients with Cerebral Palsy and Healthy Children: Comparison of Voluntary Motion and Electrically Evoked Motion
Source: Children (Basel). 2026 Jan 13;13(1):116. doi: 10.3390/children13010116 (PMC12839631; doi:10.3390/children13010116)
Supplement: Supplementary file 1 [file children-13-00116-s001.zip › S2.pdf]

Overview of all acquired time courses of the experiments under EMS. First column: velocity time course, second column: strain time course (magnitude of contraction component), third column: all force time courses during MRI acquisition (plantarflexion on foot pedal), fourth column: mean force time course. Plots of all experiments in n=14 pediatric cerebral palsy (CP) patients and n=13 controls (healthy, typically developing children) are shown. The patients were scanned pre, 6 weeks, and 12 weeks post BTX injection. The velocity data were acquired with the cine phase contrast MR sequences with VENC 25 cm/s and/or VENC 10 cm/s. EMS: electrical muscle stimulation, VENC: velocity encoding, BTX: botulinum toxin A.

control 1
Venc 25

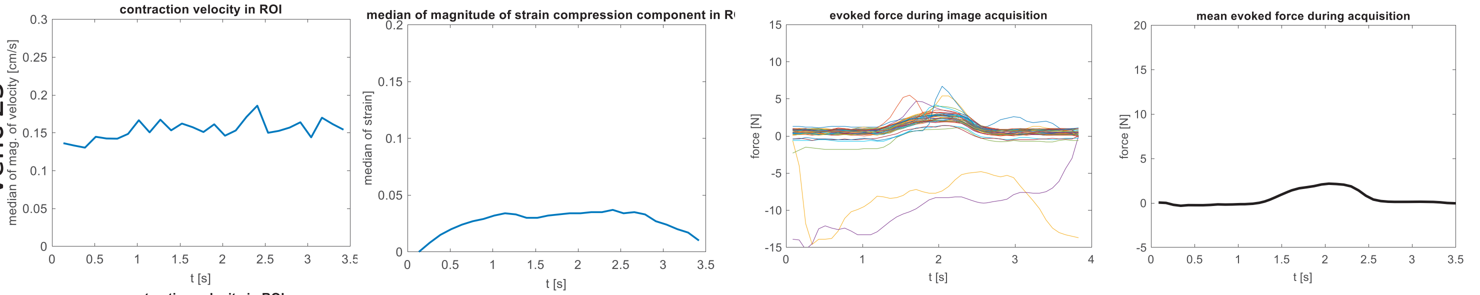

control 2
Venc 25

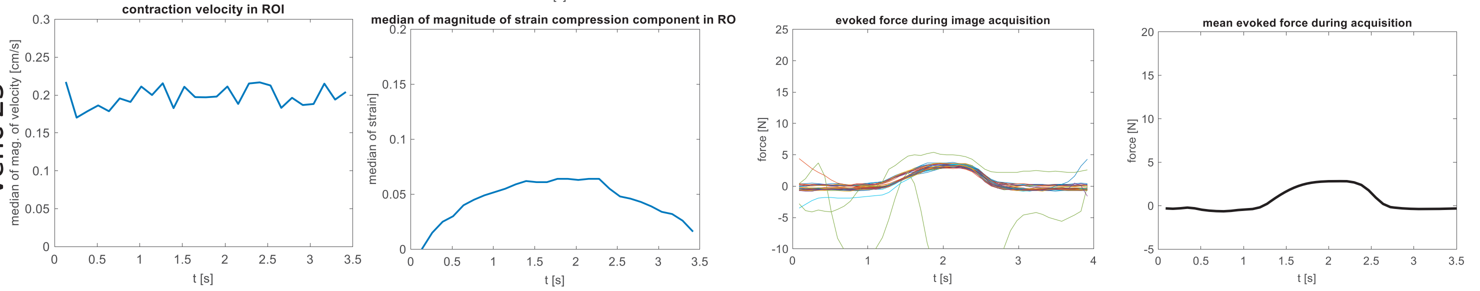

control 3

No data processing: experiment failed due to severe motion artifacts

control 4
Venc 25

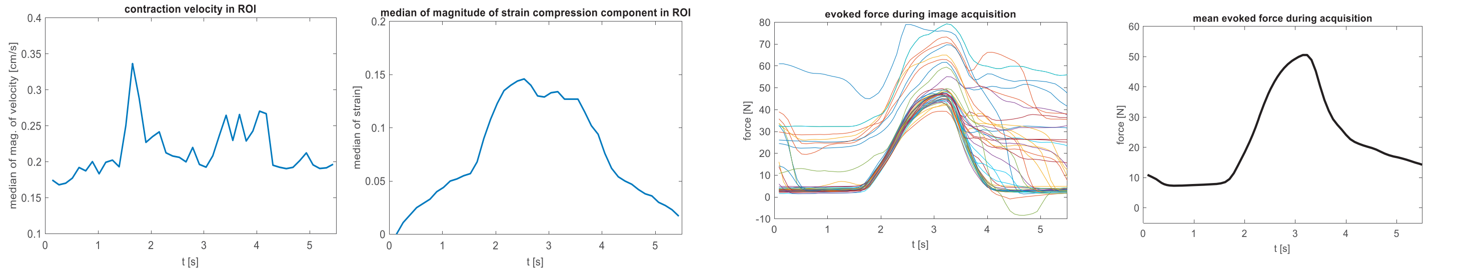

control 5
Venc 25

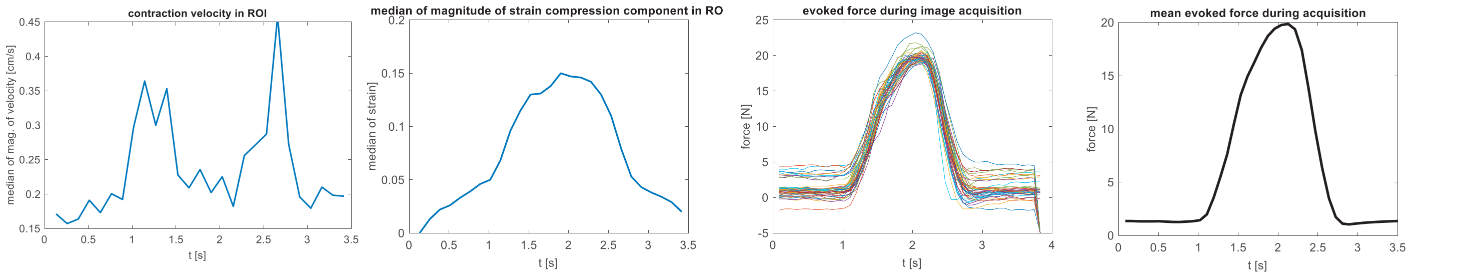

control 6
Venc 25

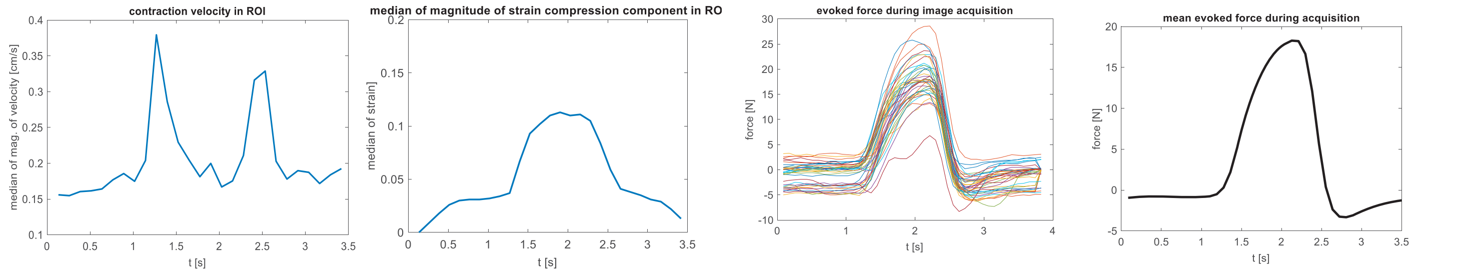

control 7
Venc 25

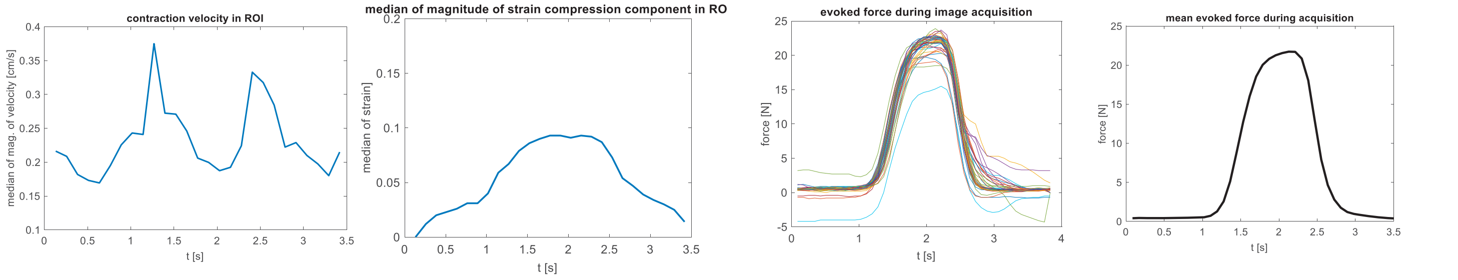

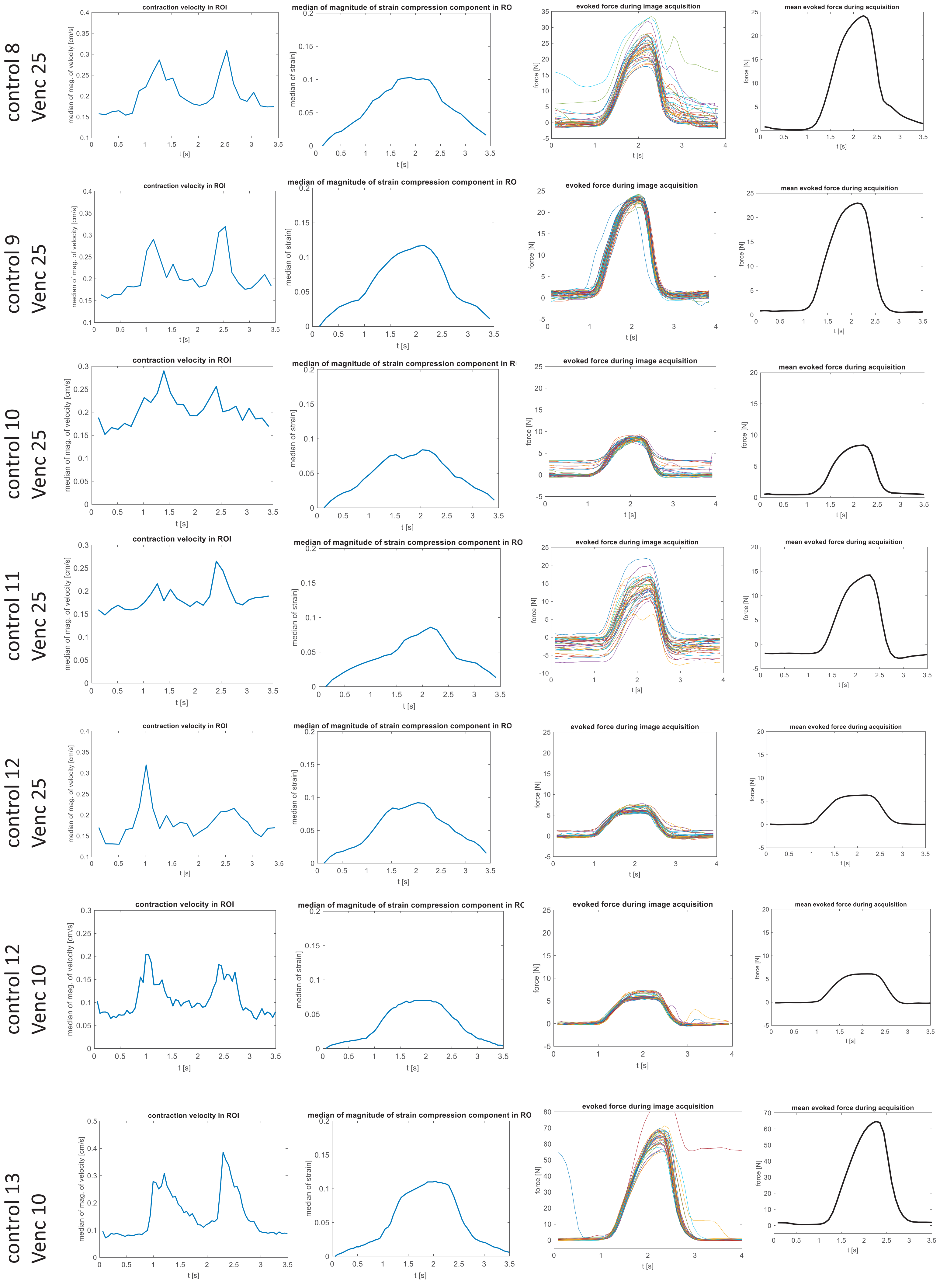
control 8
Venc 25
control 9
Venc 25
control 10
Venc 25
control 11
Venc 25
control 12
Venc 25
control 12
Venc 10
control 13
Venc 10
contraction velocity in ROI
median of mag. of velocity [cm/s]
median of magnitude of strain compression component in RO
median of strain]
evoked force during image acquisition
force [N]
mean evoked force during acquisition
t [s]

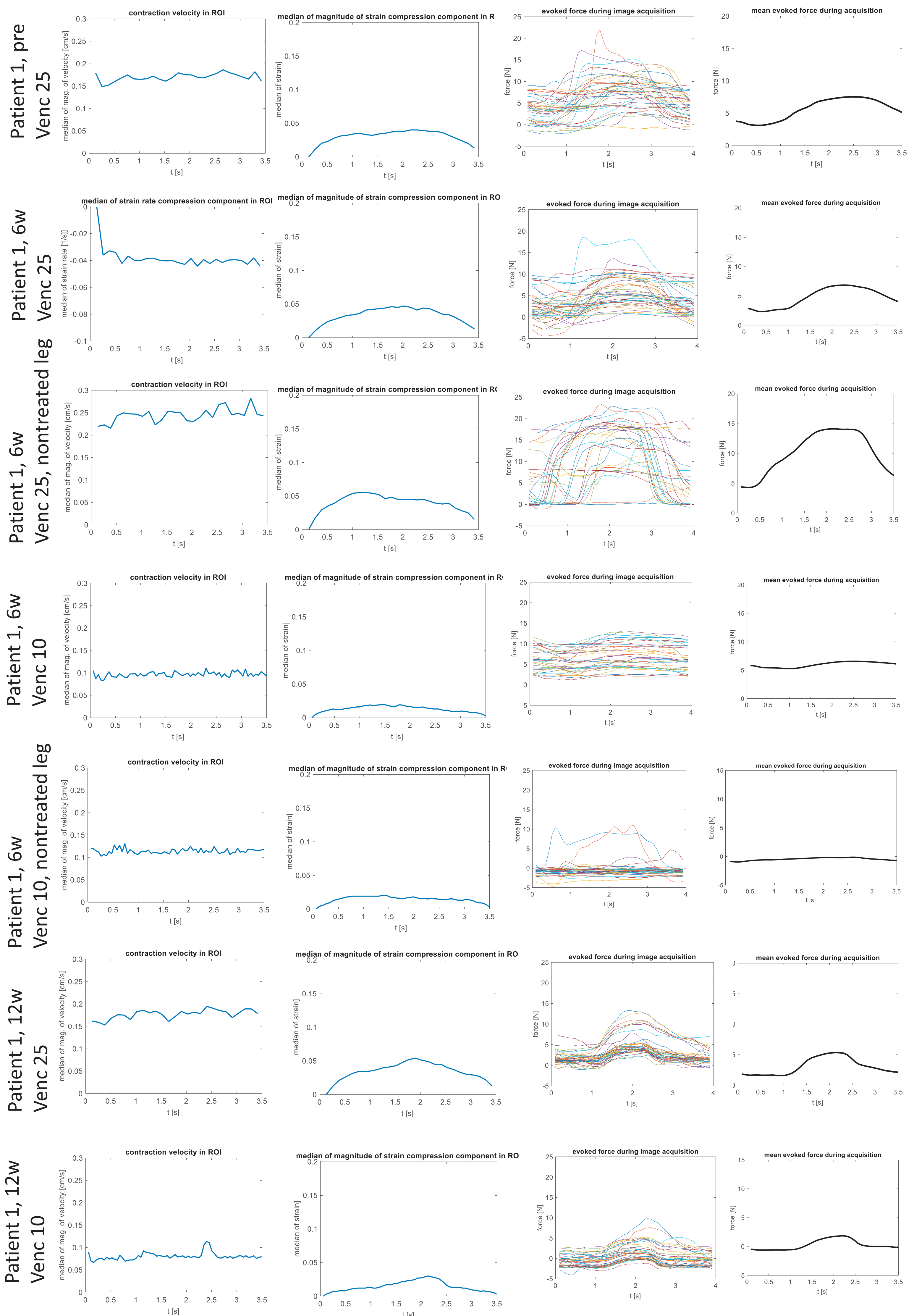
Patient 1, pre
Venc 25
contraction velocity in ROI
median of magnitude of strain compression component in R
evoked force during image acquisition
mean evoked force during acquisition
Patient 1, 6w
Venc 25
median of strain rate compression component in ROI
median of magnitude of strain compression component in RO
evoked force during image acquisition
mean evoked force during acquisition
Patient 1, 6w
Venc 25, nontreated leg
contraction velocity in ROI
median of magnitude of strain compression component in R
evoked force during image acquisition
mean evoked force during acquisition
Patient 1, 6w
Venc 10
contraction velocity in ROI
median of magnitude of strain compression component in R
evoked force during image acquisition
mean evoked force during acquisition
Patient 1, 6w
Venc 10, nontreated leg
contraction velocity in ROI
median of magnitude of strain compression component in R
evoked force during image acquisition
mean evoked force during acquisition
Patient 1, 12w
Venc 25
contraction velocity in ROI
median of magnitude of strain compression component in RO
evoked force during image acquisition
mean evoked force during acquisition
Patient 1, 12w
Venc 10
contraction velocity in ROI
median of magnitude of strain compression component in RO
evoked force during image acquisition
mean evoked force during acquisition
median of mag. of velocity [cm/s]
median of strain rate [1/s]
median of strain]
force [N]
t [s]

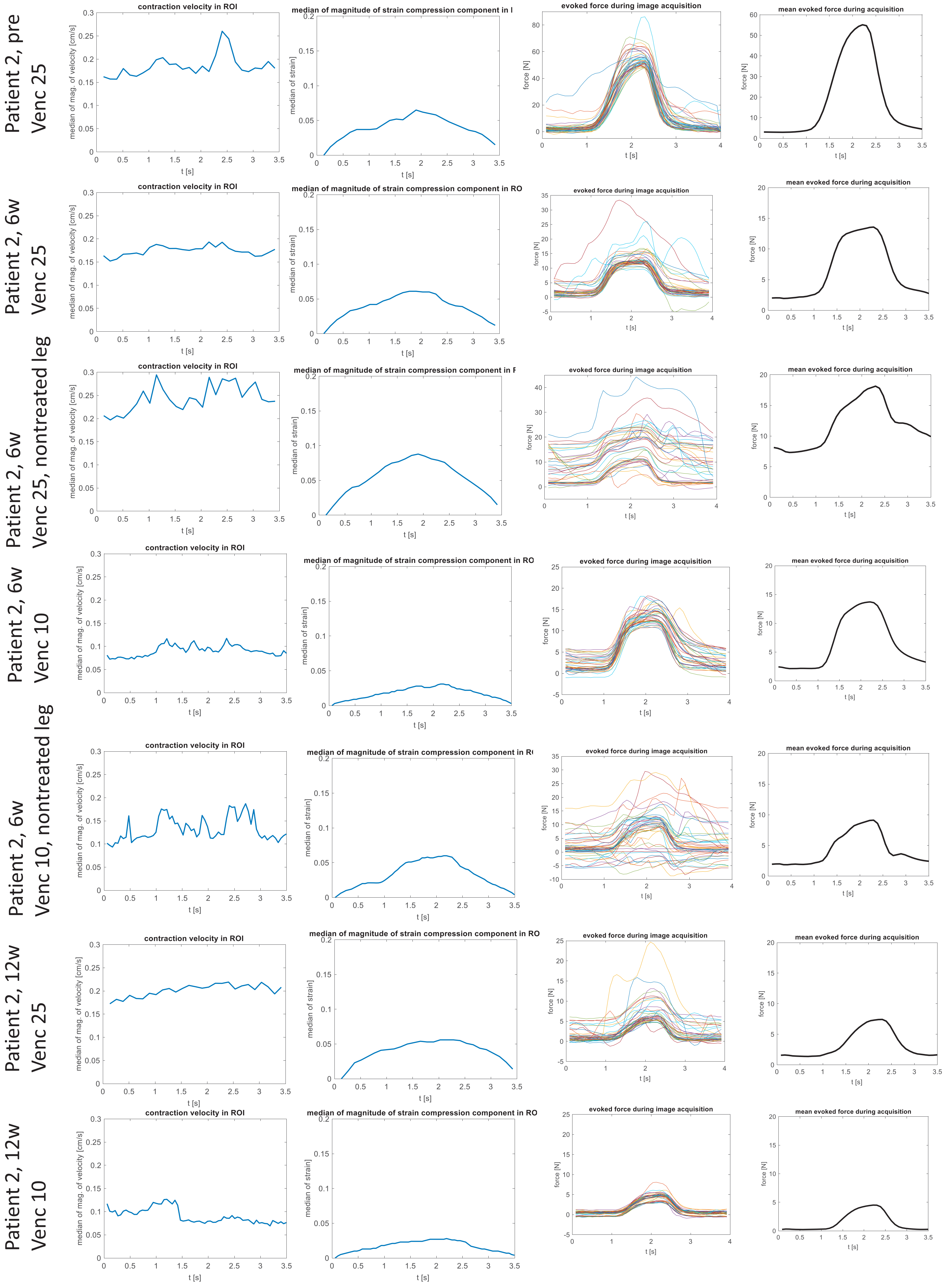

Patient 2, pre
Venc 25
Patient 2, 6w
Venc 25
Patient 2, 6w
Venc 25, nontreated leg
Patient 2, 6w
Venc 10
Patient 2, 6w
Venc 10, nontreated leg
Patient 2, 12w
Venc 25
Patient 2, 12w
Venc 10
contraction velocity in ROI
median of mag. of velocity [cm/s]
median of magnitude of strain compression component in ROI
median of strain]
evoked force during image acquisition
mean evoked force during acquisition
force [N]
t [s]

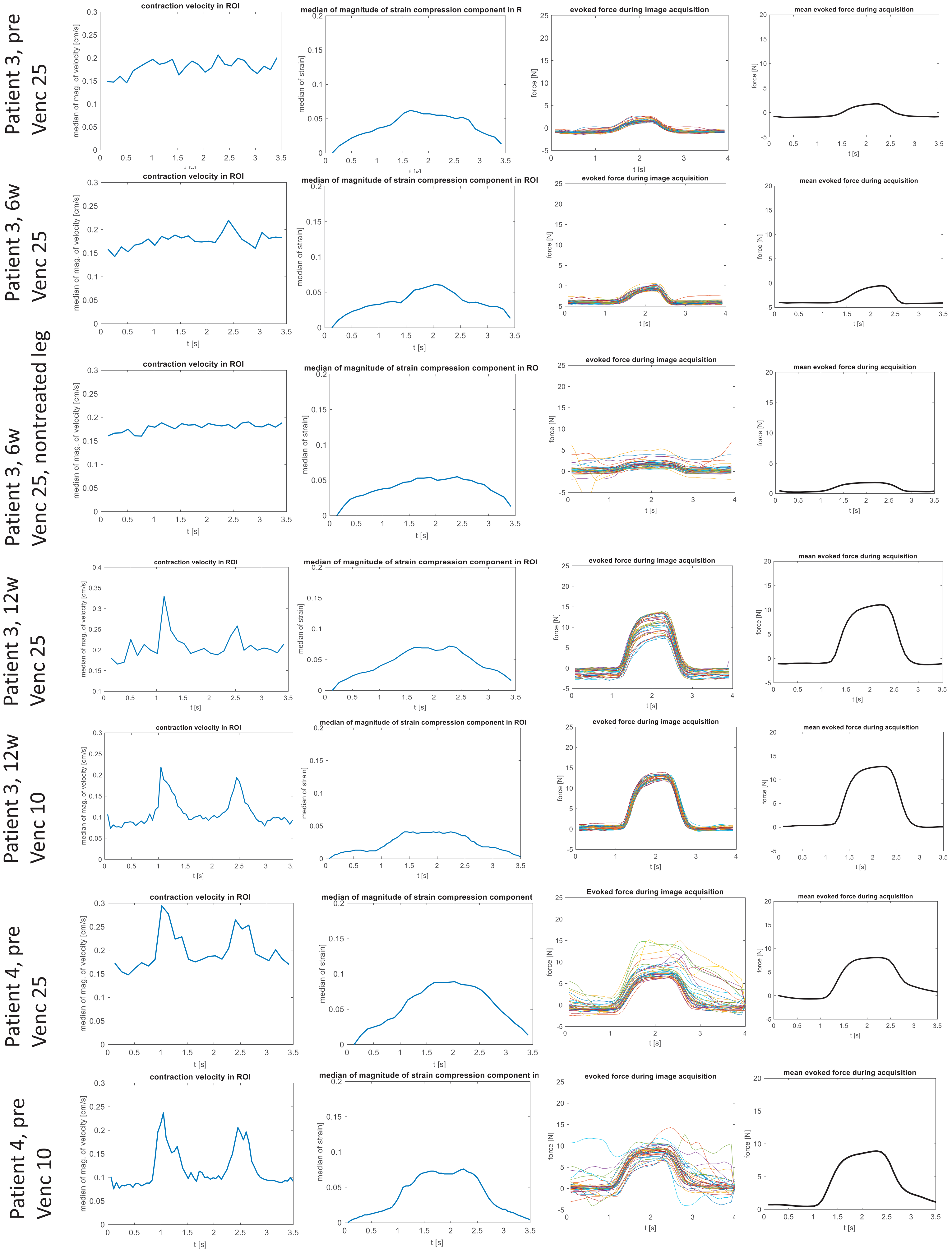
Patient 3, pre Venc 25
Patient 3, 6w Venc 25
Patient 3, 6w Venc 25, nontreated leg
Patient 3, 12w Venc 25
Patient 3, 12w Venc 10
Patient 4, pre Venc 25
Patient 4, pre Venc 10
contraction velocity in ROI
median of mag. of velocity [cm/s]
median of magnitude of strain compression component in ROI
median of strain]
evoked force during image acquisition
mean evoked force during acquisition
force [N]
t [s]

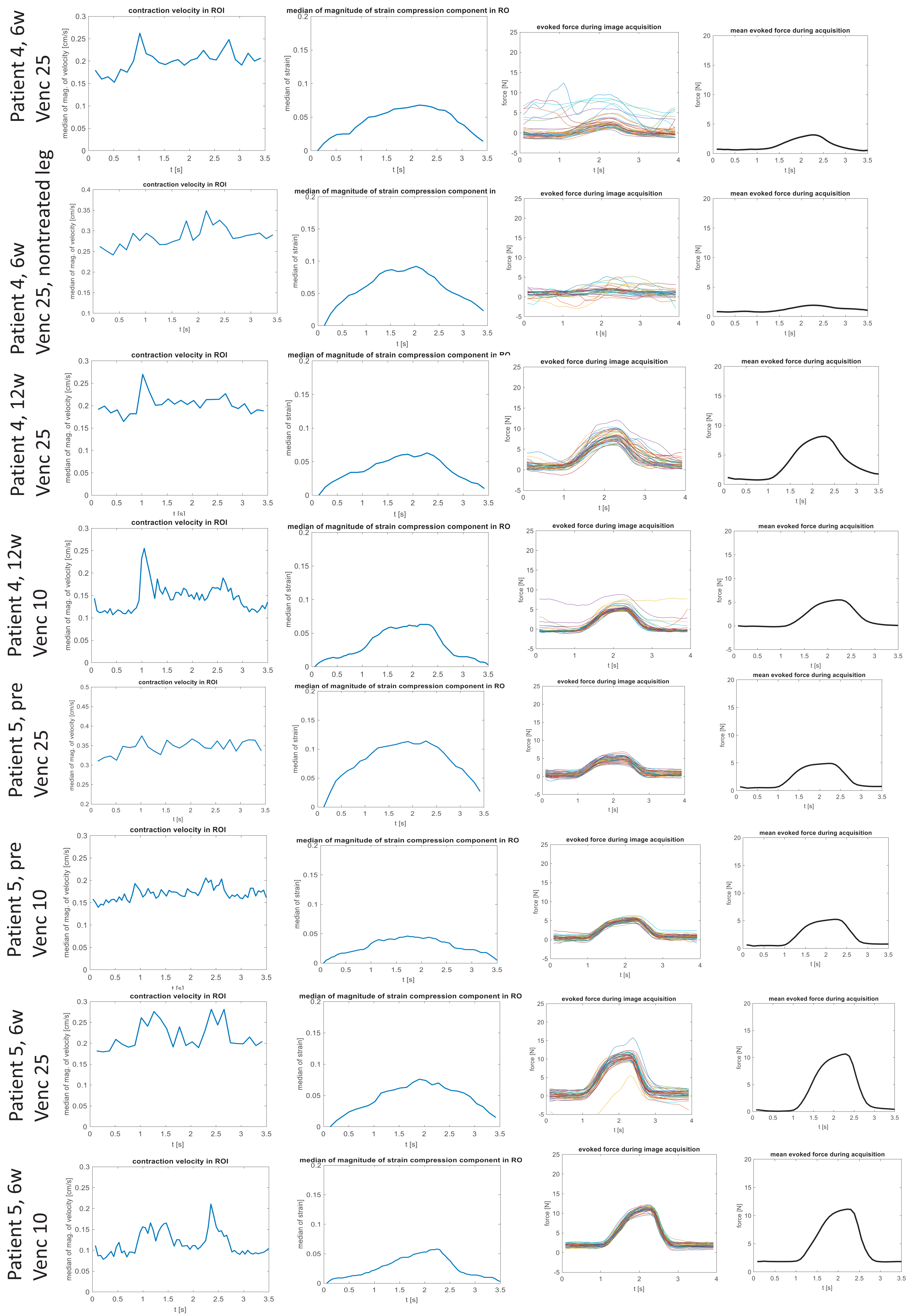
Patient 4, 6w
Venc 25
Patient 4, 6w
Venc 25, nontreated leg
Patient 4, 12w
Venc 25
Patient 4, 12w
Venc 10
Patient 5, pre
Venc 25
Patient 5, pre
Venc 10
Patient 5, 6w
Venc 25
Patient 5, 6w
Venc 10
contraction velocity in ROI
median of mag. of velocity [cm/s]
median of magnitude of strain compression component in RO
median of strain]
evoked force during image acquisition
force [N]
mean evoked force during acquisition
t [s]

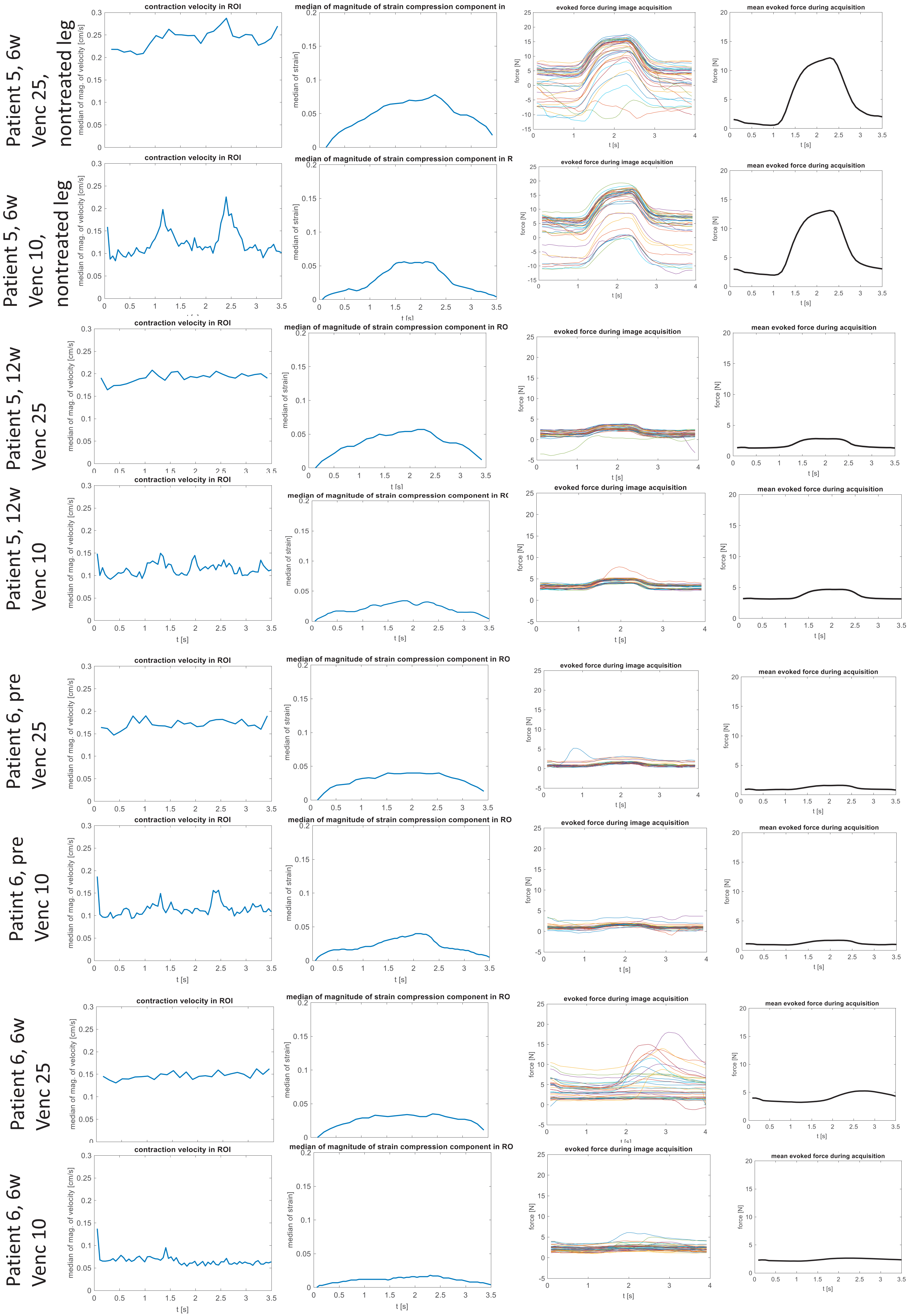

Patient 5, 6w
Venc 25,
nontreated leg
contraction velocity in ROI
median of mag. of velocity [cm/s]
median of magnitude of strain compression component in
median of strain]
evoked force during image acquisition
force [N]
t [s]
mean evoked force during acquisition
force [N]
t [s]
Patient 5, 6w
Venc 10,
nontreated leg
contraction velocity in ROI
median of mag. of velocity [cm/s]
median of magnitude of strain compression component in R
median of strain]
evoked force during image acquisition
force [N]
t [s]
mean evoked force during acquisition
force [N]
t [s]
Patient 5, 12w
Venc 25
contraction velocity in ROI
median of mag. of velocity [cm/s]
median of magnitude of strain compression component in RO
median of strain]
t [s]
evoked force during image acquisition
force [N]
t [s]
mean evoked force during acquisition
force [N]
t [s]
Patient 5, 12w
Venc 10
contraction velocity in ROI
median of mag. of velocity [cm/s]
t [s]
median of magnitude of strain compression component in RO
median of strain]
t [s]
evoked force during image acquisition
force [N]
t [s]
mean evoked force during acquisition
force [N]
t [s]
Patient 6, pre
Venc 25
contraction velocity in ROI
median of mag. of velocity [cm/s]
t [s]
median of magnitude of strain compression component in RO
median of strain]
evoked force during image acquisition
force [N]
t [s]
mean evoked force during acquisition
force [N]
t [s]
Patint 6, pre
Venc 10
contraction velocity in ROI
median of mag. of velocity [cm/s]
t [s]
median of magnitude of strain compression component in RO
median of strain]
t [s]
evoked force during image acquisition
force [N]
t [s]
mean evoked force during acquisition
force [N]
t [s]
Patient 6, 6w
Venc 25
contraction velocity in ROI
median of mag. of velocity [cm/s]
median of magnitude of strain compression component in RO
median of strain]
evoked force during image acquisition
force [N]
mean evoked force during acquisition
force [N]
t [s]
Patient 6, 6w
Venc 10
contraction velocity in ROI
median of mag. of velocity [cm/s]
t [s]
median of magnitude of strain compression component in RO
median of strain]
t [s]
evoked force during image acquisition
force [N]
t [s]
mean evoked force during acquisition
force [N]
t [s]

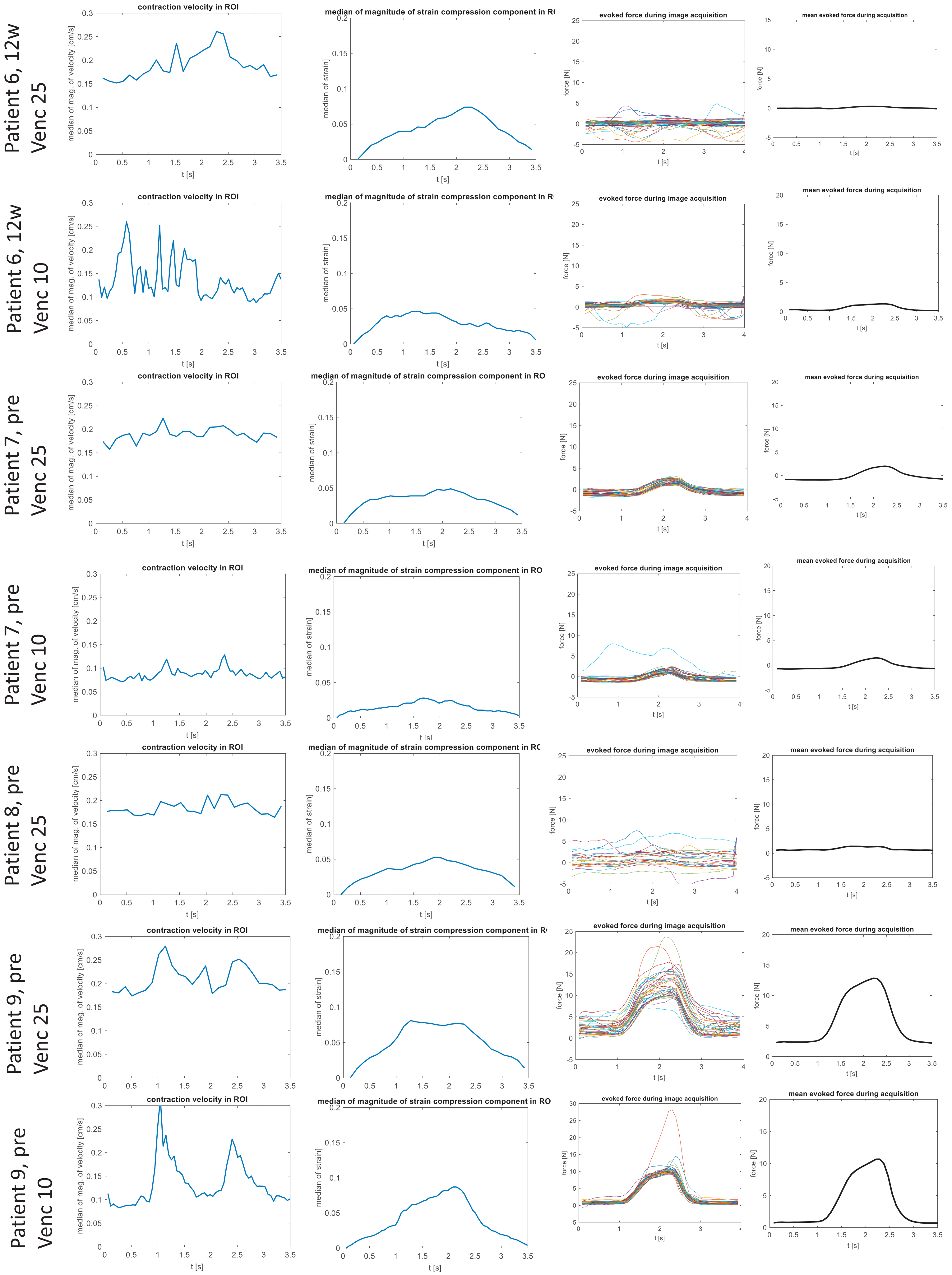
Patient 6, 12w
Venc 25
contraction velocity in ROI
median of mag. of velocity [cm/s]
t [s]
median of magnitude of strain compression component in R
median of strain]
evoked force during image acquisition
force [N]
mean evoked force during acquisition
Patient 6, 12w
Venc 10
Patient 7, pre
Venc 25
median of magnitude of strain compression component in RO
Patient 7, pre
Venc 10
Patient 8, pre
Venc 25
Patient 9, pre
Venc 25
Patient 9, pre
Venc 10

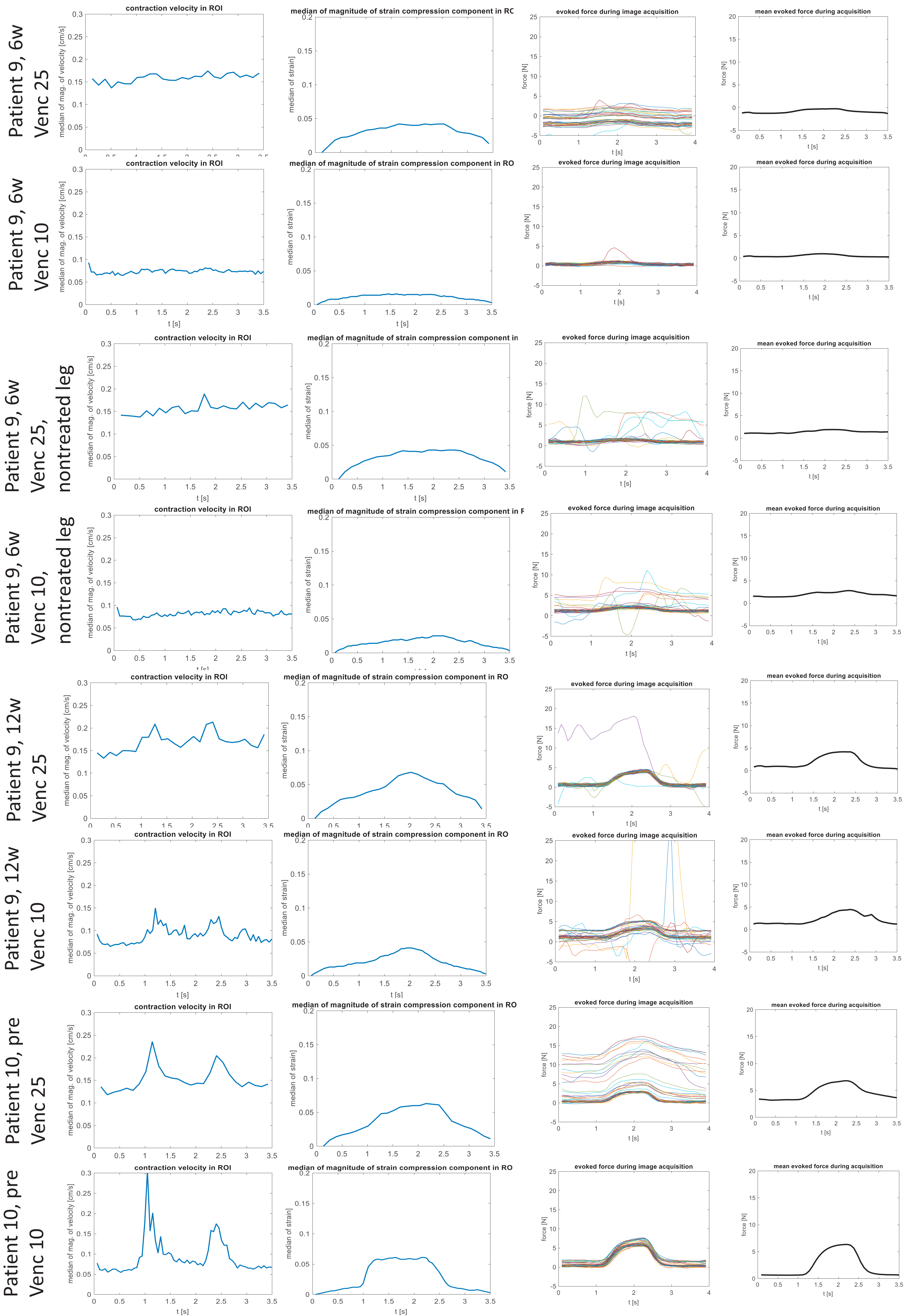
Patient 9, 6w
Venc 25
Patient 9, 6w
Venc 10
Patient 9, 6w
Venc 25,
nontreated leg
Patient 9, 6w
Venc 10,
nontreated leg
Patient 9, 12w
Venc 25
Patient 9, 12w
Venc 10
Patient 10, pre
Venc 25
Patient 10, pre
Venc 10
contraction velocity in ROI
median of mag. of velocity [cm/s]
median of magnitude of strain compression component in RO
median of strain]
evoked force during image acquisition
force [N]
mean evoked force during acquisition
t [s]

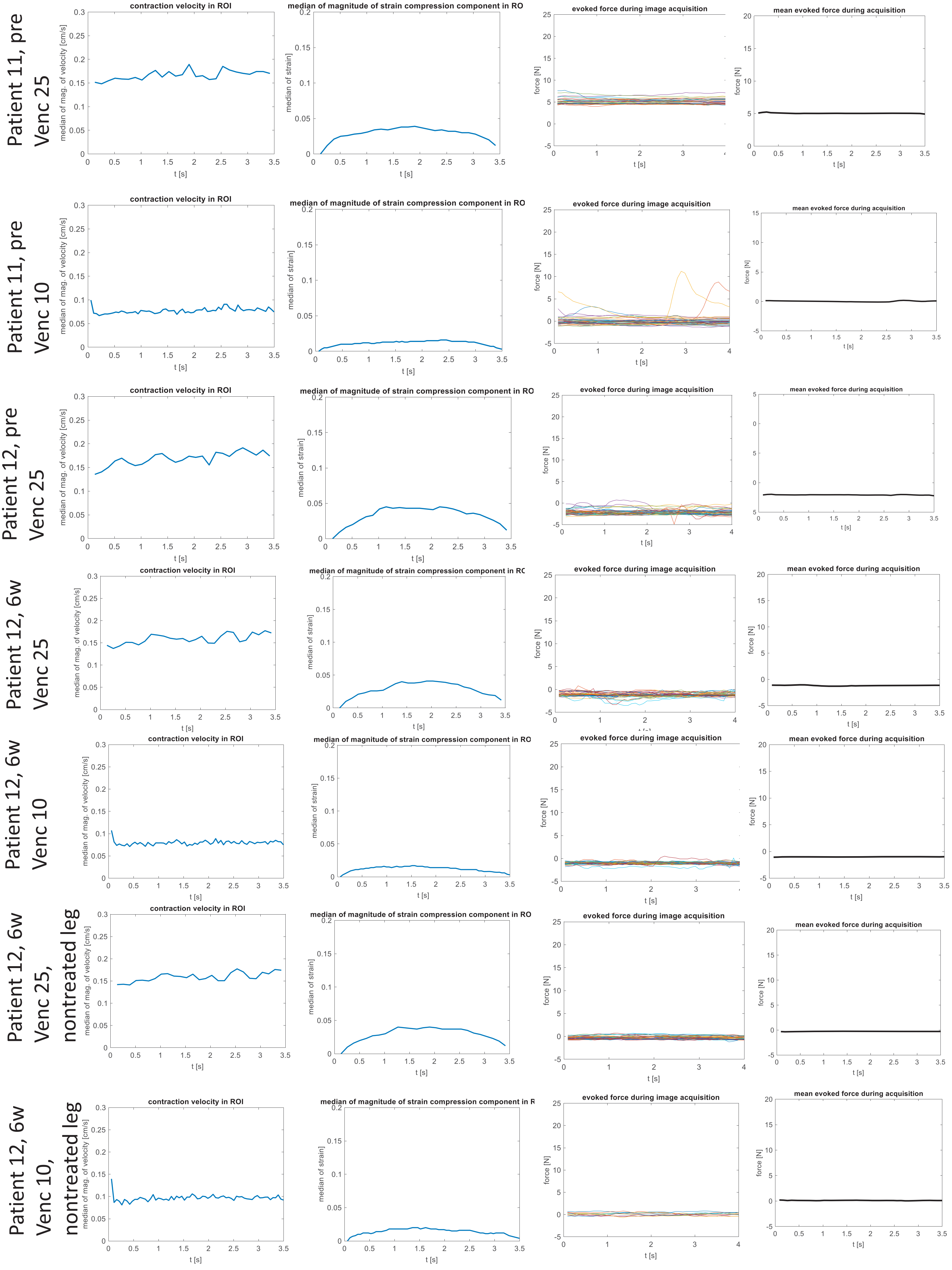

Patient 11, pre Venc 25
Patient 11, pre Venc 10
Patient 12, pre Venc 25
Patient 12, 6w Venc 25
Patient 12, 6w Venc 10
Patient 12, 6w Venc 25, nontreated leg
Patient 12, 6w Venc 10, nontreated leg
contraction velocity in ROI
median of mag. of velocity [cm/s]
median of magnitude of strain compression component in RO
median of strain]
evoked force during image acquisition
mean evoked force during acquisition
force [N]
t [s]

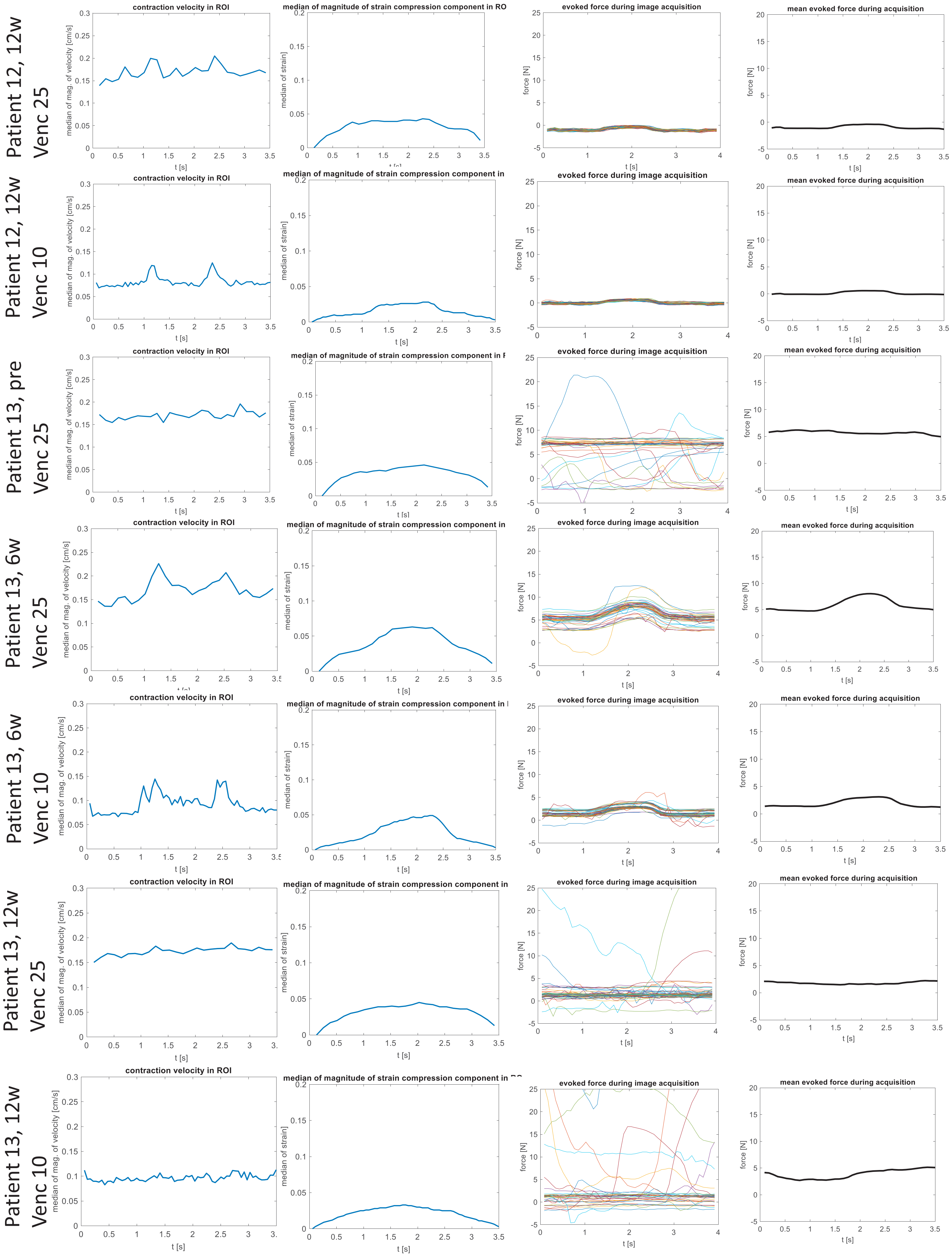

Patient 12, 12w
Venc 25
Patient 12, 12w
Venc 10
Patient 13, pre
Venc 25
Patient 13, 6w
Venc 25
Patient 13, 6w
Venc 10
Patient 13, 12w
Venc 25
Patient 13, 12w
Venc 10
contraction velocity in ROI
median of mag. of velocity [cm/s]
median of magnitude of strain compression component in RO
median of strain]
evoked force during image acquisition
mean evoked force during acquisition
force [N]
t [s]

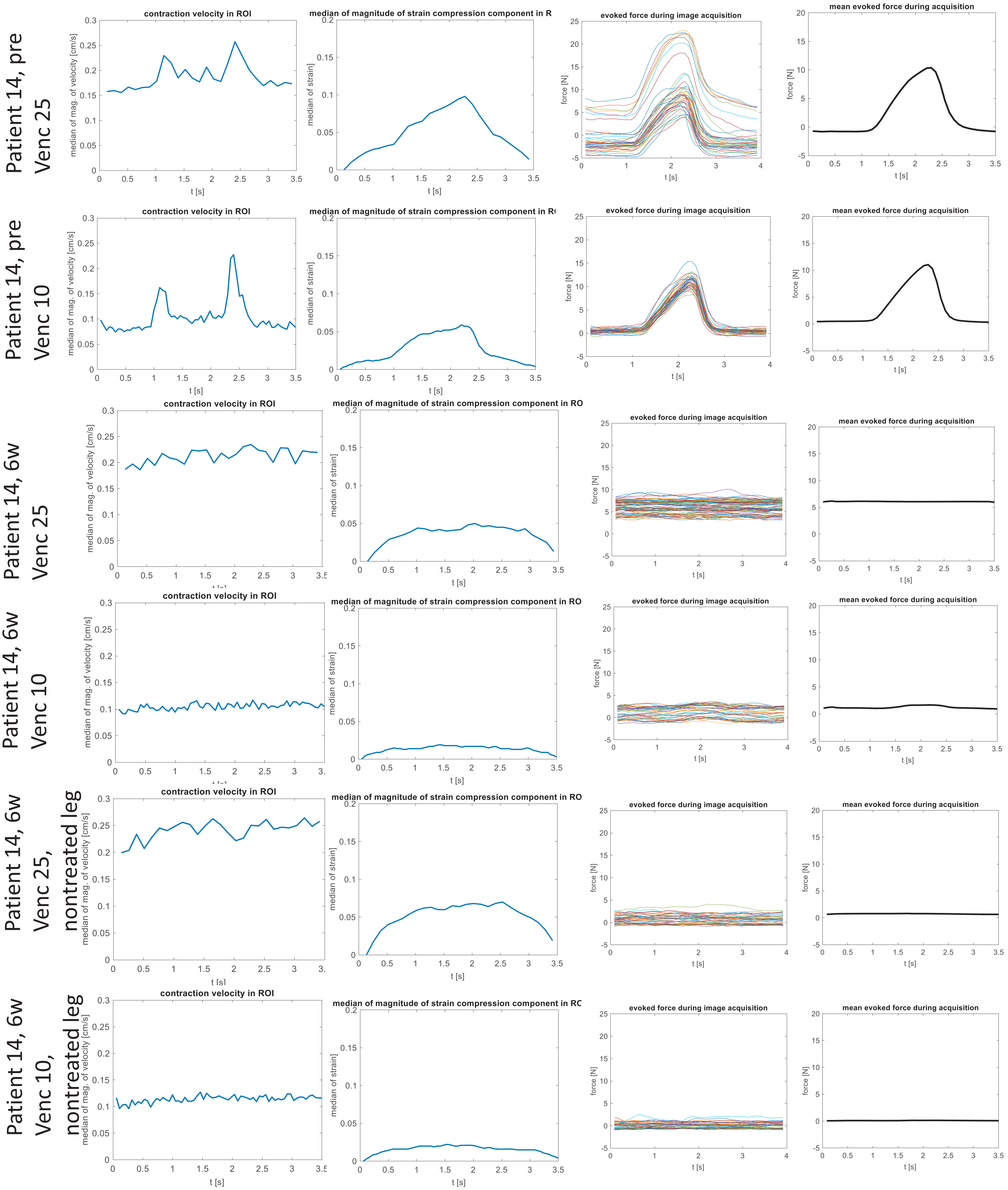

Patient 14, pre Venc 25
Patient 14, pre Venc 10
Patient 14, 6w Venc 25
Patient 14, 6w Venc 10
Patient 14, 6w Venc 25, nontreated leg
Patient 14, 6w Venc 10, nontreated leg
contraction velocity in ROI
median of mag. of velocity [cm/s]
median of magnitude of strain compression component in R
median of strain]
evoked force during image acquisition
force [N]
mean evoked force during acquisition
t [s]
