# Supplementary material for: Cine Phase Contrast Magnetic Resonance Imaging of Calf Muscle Contraction in Pediatric Patients with Cerebral Palsy and Healthy Children: Comparison of Voluntary Motion and Electrically Evoked Motion
Source: Children (Basel). 2026 Jan 13;13(1):116. doi: 10.3390/children13010116 (PMC12839631; doi:10.3390/children13010116)
Supplement: Supplementary file 1 [file children-13-00116-s001.zip › S3.pdf]

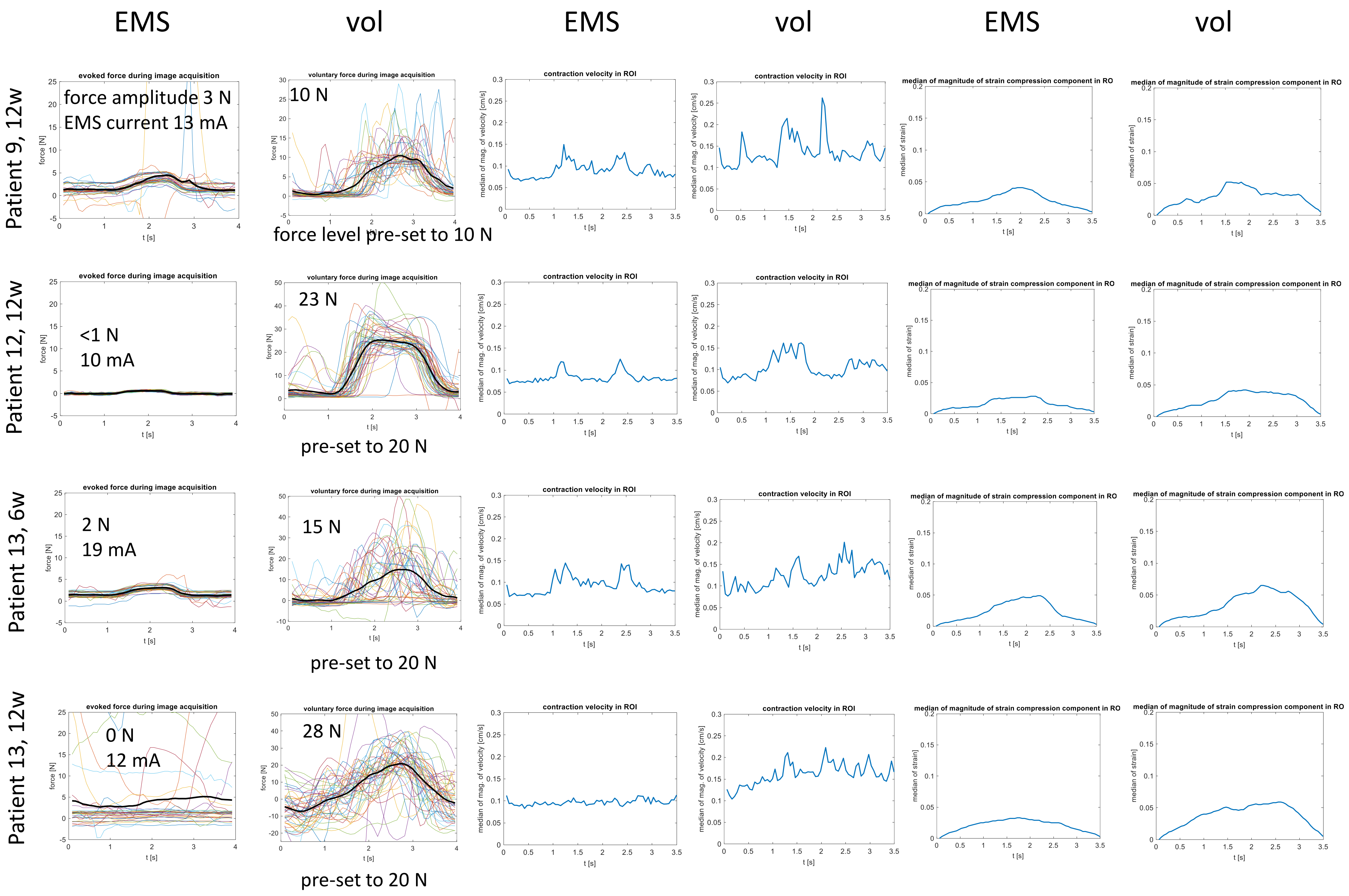
EMS
vol
EMS
vol
EMS
vol
Patient 9, 12w
evoked force during image acquisition
force amplitude 3 N
EMS current 13 mA
voluntary force during image acquisition
10 N
force level pre-set to 10 N
contraction velocity in ROI
median of magnitude of strain compression component in RO
Patient 12, 12w
<1 N
10 mA
23 N
pre-set to 20 N
Patient 13, 6w
2 N
19 mA
15 N
pre-set to 20 N
Patient 13, 12w
0 N
12 mA
28 N
pre-set to 20 N
force [N]
t [s]
median of mag. of velocity [cm/s]
median of strain]

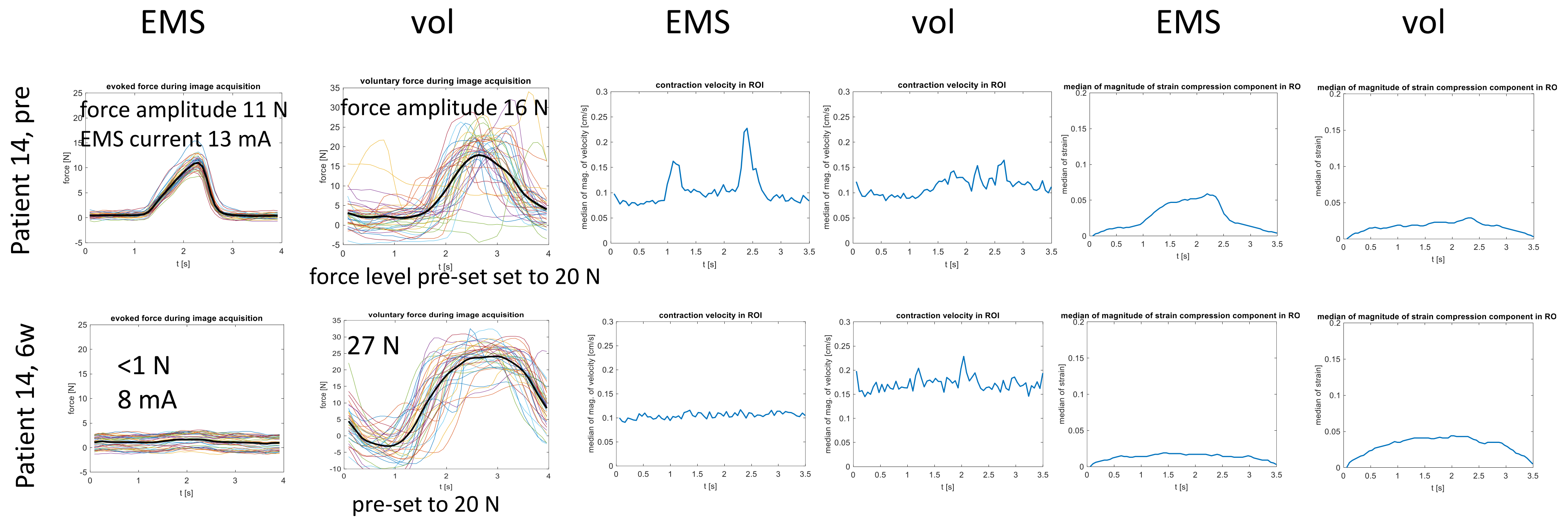
EMS
vol
EMS
vol
EMS
vol
Patient 14, pre
evoked force during image acquisition
force amplitude 11 N
EMS current 13 mA
force [N]
t [s]
voluntary force during image acquisition
force amplitude 16 N
force [N]
t [s]
force level pre-set set to 20 N
contraction velocity in ROI
median of mag. of velocity [cm/s]
t [s]
contraction velocity in ROI
median of mag. of velocity [cm/s]
t [s]
median of magnitude of strain compression component in RO
median of strain]
t [s]
median of magnitude of strain compression component in RO
median of strain]
t [s]
Patient 14, 6w
evoked force during image acquisition
<1 N
8 mA
force [N]
t [s]
voluntary force during image acquisition
27 N
force [N]
t [s]
pre-set to 20 N
contraction velocity in ROI
median of mag. of velocity [cm/s]
t [s]
contraction velocity in ROI
median of mag. of velocity [cm/s]
t [s]
median of magnitude of strain compression component in RO
median of strain]
t [s]
median of magnitude of strain compression component in RO
median of strain]
t [s]

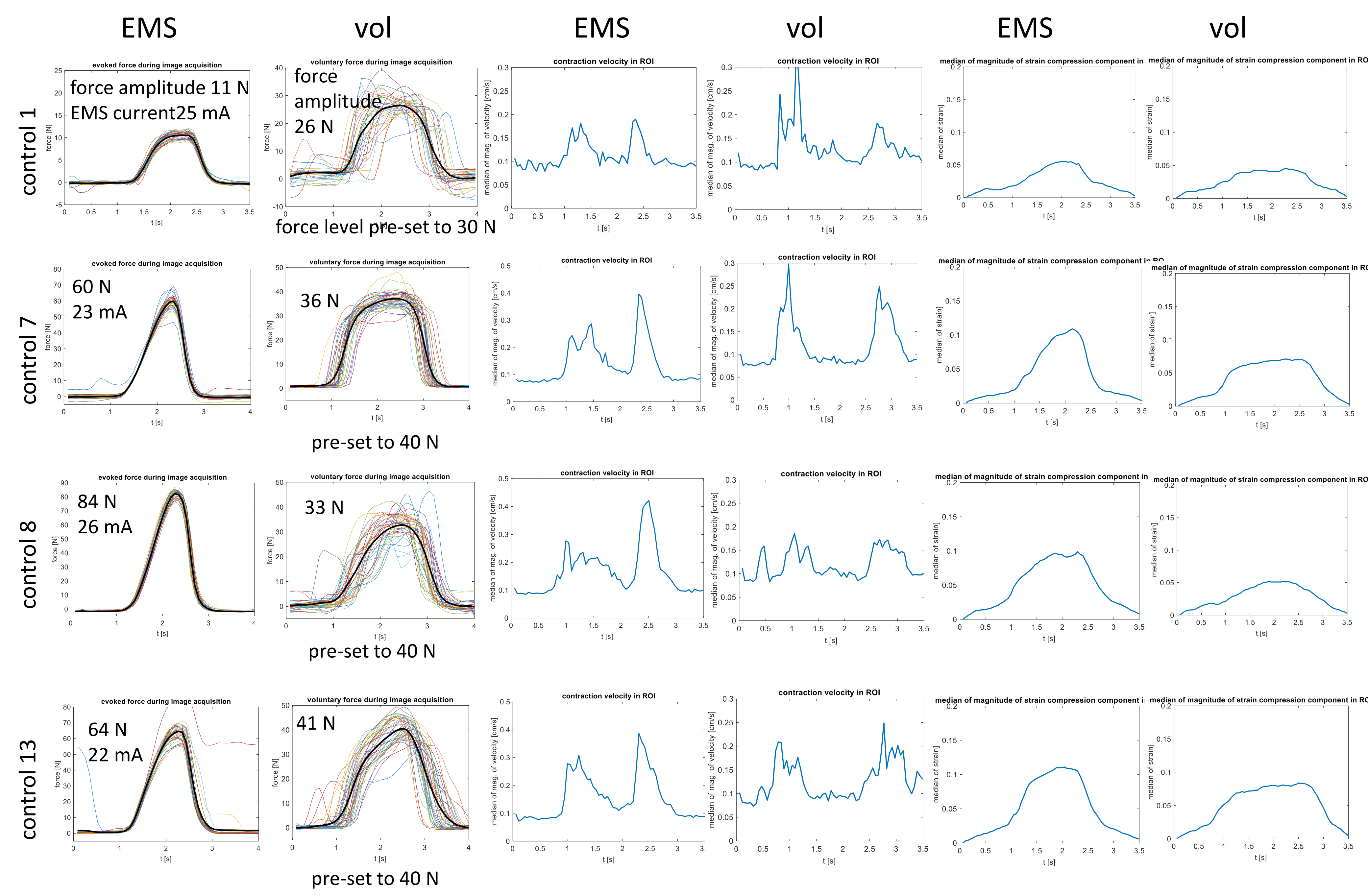
EMS
vol
EMS
vol
EMS
vol
control 1
evoked force during image acquisition
force amplitude 11 N
EMS current25 mA
voluntary force during image acquisition
force amplitude 26 N
force level pre-set to 30 N
contraction velocity in ROI
median of magnitude of strain compression component in
control 7
60 N
23 mA
36 N
pre-set to 40 N
control 8
84 N
26 mA
33 N
pre-set to 40 N
control 13
64 N
22 mA
41 N
pre-set to 40 N
force [N]
t [s]
median of mag. of velocity [cm/s]
median of strain]

**Comparison of the results under EMS and voluntary motion.** All the force plots of the plantarflexion on the foot pedal, velocity time courses, and strain time courses of the cine phase contrast MR sequences (VENC 10 cm/s) for all the four pediatric cerebral palsy (CP) patients and the four controls (healthy, typically developing children) that performed the voluntary paradigm experiments are shown. The amplitude of the EMS current (in mA), the pre-set force amplitude of the voluntary motion paradigm (in N), and the mean force amplitudes (in N) are stated in the graphs. The mean force is shown in black. For the strain time courses the magnitude of the contraction component is shown. The patients were scanned pre, 6 weeks, and 12 weeks post BTX injection. EMS: electrical muscle stimulation, vol: voluntary motion, VENC: velocity encoding, BTX: botulinum toxin A.
