# Supplementary material for: Cine Phase Contrast Magnetic Resonance Imaging of Calf Muscle Contraction in Pediatric Patients with Cerebral Palsy and Healthy Children: Comparison of Voluntary Motion and Electrically Evoked Motion
Source: Children (Basel). 2026 Jan 13;13(1):116. doi: 10.3390/children13010116 (PMC12839631; doi:10.3390/children13010116)
Supplement: Supplementary file 1 [file children-13-00116-s001.zip › S4.pdf]

patient number	max. voluntary force [N]			
	pre BTX	6w post BTX	6w post BTX, non-treated leg	12w post BTX
1	94	108	182	113
2	258	129	342	254
3	121	108	131	96
4	64	12	114	84
5	46	70	72	76
6	36	65	100	40
7	92			
8	143			
9	127	208	253	199
10	177			
11	71			
12	157	163	218	219
13	172	152	286	135
14	104	83	108	

control number	max. voluntary force [N]	
	1st session	2nd session
1	275	273
2	388	
3	326	
4	292	
5	257	
6	260	
7	151	273
8	273	268
9	155	
10	170	
11	108	
12	207	
13	343	

Maximum voluntary force for plantarflexion in pediatric CP patients and healthy, typically developing children (controls). Patient force measurements were performed pre, 6 weeks, and 12 weeks post botulinum toxin A (BTX) injection.
